# Comparative chloroplast genome analyses provide insights into evolutionary history of Rhizophoraceae mangroves

**DOI:** 10.7717/peerj.16400

**Published:** 2023-11-17

**Authors:** Ying Zhang, Yuchen Yang, Meng He, Ziqi Wei, Xi Qin, Yuanhao Wu, Qingxing Jiang, Yufeng Xiao, Yong Yang, Wei Wang, Xiang Jin

**Affiliations:** 1 Hainan Academy of Forestry, Hainan Mangrove Research Institute, Haikou, Hainan, China; 2 Qiongtai Normal University, Research Center for Wild Animal and Plant Resource Protection and Utilization, Haikou, Hainan, China; 3 Lingnan Normal University, Life Science and Technology School, Zhanjiang, Guangdong, China; 4 State Key Laboratory of Biocontrol, School of Ecology, Sun Yat-sen University, Shenzhen, Guangdong, China; 5 Hainan Normal University, Ministry of Education Key Laboratory for Ecology of Tropical Islands, Key Laboratory of Tropical Animal and Plant Ecology of Hainan Province, College of Life Sciences, Haikou, Hainan, China

**Keywords:** Mangroves, Rhizophoraceae, Chloroplast genome, Organellar phylogenomics, Divergence time

## Abstract

**Background:**

The Rhizophoraceae family comprises crucial mangrove plants that inhabit intertidal environments. In China, eight Rhizophoraceae mangrove species exist. Although complete chloroplast (Cp) genomes of four Rhizophoraceae mangrove plants have been reported, the Cp genomes of the remaining four species remain unclear, impeding a comprehensive understanding of the evolutionary history of this family.

**Methods:**

Illumina high-throughput sequencing was employed to obtain the DNA sequences of Rhizophoraceae species. Cp genomes were assembled by NOVOPlasty and annotated using CpGAVAS software. Phylogenetic and divergence time analyses were conducted using MEGA and BEAST 2 software.

**Results:**

Four novel Cp genomes of Rhizophoraceae mangrove species (*Bruguiera sexangula, Bruguiera gymnorrhiza*, *Bruguiera* × *rhynchopetala* and *Rhizophora apiculata*) were successfully assembled. The four Cp genomes ranged in length from 163,310 to 164,560 bp, with gene numbers varying from 124 to 128. The average nucleotide diversity (Pi) value of the eight Rhizophoraceae Cp genomes was 0.00596. Phylogenetic trees constructed based on the complete Cp genomes supported the monophyletic origin of Rhizophoraceae. Divergence time estimation based on the Cp genomes of representative species from Malpighiales showed that the origin of Rhizophoraceae occurred at approximately 58.54–50.02 million years ago (Mya). The divergence time within the genus *Rhizophora* (∼4.51 Mya) was much earlier than the divergence time within the genus *Bruguiera* (∼1.41 Mya), suggesting recent speciation processes in these genera. Our data provides new insights into phylogenetic relationship and evolutionary history of Rhizophoraceae mangrove plants.

## Introduction

Mangroves are community of woody plants growing in intertidal zones of tropical and subtropical coasts (Francisco et al., 2018). In 2001, Lin introduced the concept of “true mangroves”, referring to woody plants that exclusively grow in intertidal mangrove ecosystems and have specific adaptations to the marine environment, distinguishing them from “semi-mangroves”, which can grow both in intertidal zones and inland (Lin, 2001). There are 70 species of mangrove plants in 27 genera and 20 families worldwide, including 28 species belonging to 16 genera and 12 families identified in China (Spalding, Blasco & Field, 1997).

Rhizophoraceae is a family of approximately 16 genera and 120 species of trees and shrubs. In China, 12 species of Rhizophoraceae plants have been identified in six genera, which can be divided into two subtribes: Rhizophoreae and Gynotrocheae (Tomlinson, 1986; Duke, 2010; Chen et al., 2015). Rhizophoreae includes four genera and eight species, all belonging to true mangroves, while Gynotrocheae includes two genera and four species mainly distributed in noncoastal lands of South China. Rhizophoraceae species share common characteristics, such as a scalariform perforation plate in wood fiber cells and subtype PV protein-containing sieve-element plastids (Behnke, 1982). However, the taxonomic system of Rhizophoraceae is under dispute due to great differences in their ecological environment, resulting in quite different morphological and anatomical structures of Rhizophoraceae plants (Geh & Keng, 1974; van Vliet, 1976a). Previously, molecular evidence has shown that Rhizophoraceae is a monophyletic group (Xu et al., 2017b; Xu et al., 2017a).

In China, the four genera of the subtribe Rhizophoreae: *Bruguiera*, *Ceriops*, *Kandelia* and *Rhizophora*, are mainly distributed in the tropical coastal areas of Hainan, Guangdong, Guangxi, Fujian, Taiwan and Hong Kong (Zhang, Zhong & Yuan, 2019). Natural hybridization events were found in *Rhizophora* and *Bruguiera* (Kathiresan, 1999; Ragavan et al., 2015), making it difficult to clarify their taxonomic relationships. Two hybrids were identified in China, *Bruguiera* × *rhynchopetala* and *Rhizophora* × *lamarckii* (Sun & Lo, 2011; Luo et al., 2017). Chloroplasts (Cp) are important organelles for plants, playing a crucial role in photosynthesis and adaptation to saline environments. Chloroplast ultrastructure and expression of chloroplast-derived proteins were found to be changed under salt stress (Daniell et al., 2016; Bose et al., 2017; Munns et al., 2020). Cp genomes have been used for comparative evolutionary research in plants due to their small sizes (normally ranging from 120 to 180 kb), well-characterized structures (two copies of inverted repeats (IRs) and large single-copy (LSC) and small single-copy (SSC) regions, including 110 to 130 genes) and slow rates of nucleotide substitution (Cavalier-Smith, 2002). Cp genome sequences can also be used for divergence time estimation of related plant species when combined with molecular clock theory and fossil records (Hohmann et al., 2015).

Four Cp genomes of Rhizophoraceae mangrove species have been reported (Zhang, Zhong & Yuan, 2019; Chen et al., 2019; Li et al., 2019; Yang et al., 2019). In this study, we assembled the complete Cp genomes of the other four Rhizophoreae species, *B. sexangula*, *B. gymnorrhiza*, *B.* × *rhynchopetala* and *R. apiculate*. Cp genome structures, sequence diversities and species divergence times were analyzed. Moreover, evolutionary analysis based on the molecular clock was used to estimate the divergence times between Rhizophoraceae and other Malpighiales plants. Our data provide comprehensive information for a better understanding of Cp genome evolution in Rhizophoraceae mangrove species.

## Materials & Methods

### Plant sampling, DNA preparation and Illumina sequencing

Four Rhizophoraceae mangrove leaf samples were collected from Dongzhai Harbor National Natural Reserve, Haikou, China (20°17′N, 110°35′E). The corresponding voucher specimens of *B. gymnorrhiza*, *B. sexangula*, *B.* × *rhynchopetala* and *R. apiculata* were deposited in the Hainan Normal University herbarium (BG-001, BS-001, BR-001 and RA-001). Yong Yang collected the specimens and Ying Zhang identified them with the permission of the authority of Dongzhai Harbor Mangrove Natural Reserve. All experiments on the plant material complied with the plant research guidelines of Dongzhai Harbor Mangrove Natural Reserve. The DNA extraction was carried out according to previous research (Yang et al., 2019). Then, DNA was sent to HTSW (Shenzhen, China) and sequenced using an Illumina HiSeq-2000 platform with 100 bp paired-end reads following the manufacturer’s instructions.

### Chloroplast genome assembly, annotation, alignment, and visualization

Raw reads were filtered by cutadapt and fastp software to remove adapter sequences and low-quality reads. Then, fastqc (http://www.bioinformatics.babraham.ac.uk/projects/fastqc/) was used to evaluate the sequencing read quality. NOVOPlasty (Dierckxsens, Mardulyn & Smits, 2016) was used to perform the initial assembly, and the Cp genome sequence of *R. stylosa* (Genbank accession number NC_042819.1) and *R. mucronata* (MZ959046) were used as references. The assembled Cp genome sequences were annotated by CpGAVAS with default parameters (Liu et al., 2012). The circular maps were drawn using OGDRAW (Greiner, Lehwark & Bock, 2019).

DnaSP6 (Rozas et al., 2017) was used to calculate the nucleotide diversity (Pi) and to detect highly variable sites among the eight Rhizophoraceae Cp genomes with a window length of 600 bp and a step size of 200 bp. The Jukes-Cantor correction were used to calculate Pi. The variation in the eight complete Cp genome sequences of *B. sexangula* (MT129628), *B. gymnorrhiza* (MT129629), *B.* × *rhynchopetala* (MT129630), *R. apiculate* (MT129631), *R. stylosa* (MK070169.1), *R.* × *lamarckii* (MK392466.1), *Ceriops tagal* (MH240830.1) and *Kandelia obovata* (MH277332.1) were analyzed using mVISTA software in shuffle-LAGAN mode (Frazer et al., 2004). The borders between single copy regions (LSC and SSC) and IR regions were compared using IRscope software (Amiryousefi, Hyvönen & Poczai, 2018).

### SSR, codon frequency, and RNA editing analyses

Simple sequence repeats (SSRs) in the eight Cp genomes of Rhizophoraceae plants were detected by MISA (http://pgrc.ipk-gatersleben.de/misa/). The parameters of the software were set as follows: the minimum number of repeats for mononucleotides was 10, dinucleotides was five, trinucleotides was four, tetranucleotides was three, pentanucleotide was three and hexanucleotides was three.

The codonW software (http://codonw.sourceforge.net/) was used to investigate the codon usage patterns of all eight Rhizophoraceae mangroves studied here using default parameters. The distribution of codon usage of the eight Rhizophoraceae species was presented in a heatmap constructed by HemI 1.0 (Deng et al., 2014). RNA editing sites were predicted using Predictive RNA Editor for Plants (PREP) with a cutoff value of 0.8 (Mower, 2009).

### Phylogenetic analysis and divergence time estimation of Rhizophoraceae species within Malpighiales

The divergence time of Rhizophoraceae species in Malpighiales was first estimated. Briefly, Cp genomes were either retrieved from the NCBI or newly generated for this study. Sequences of 65 conserved Cp genes (*accD*, *atpA*, *atpB*, *atpE*, *atpH*, *atpI*, *ccsA*, *cemA*, *clpP*, *matK*, *ndhA*, *ndhB*, *ndhC*, *ndhD*, *ndhE*, *ndhF*, *ndhG*, *ndhH*, *ndhI*, *ndhJ*, *ndhK*, *petA*, *petB*, *petD*, *petG*, *petN*, *psaA*, *psaB*, *psaC*, *psaI*, *psaJ*, *psbA*, *psbB*, *psbC*, *psbD*, *psbE*, *psbF*, *psbH*, *psbI*, *psbL*, *psbM*, *psbN*, *psbT*, *psbZ*, *rbcL*, *rpl14*, *rpl16*, *rpl2*, *rpl20*, *rpl22*, *rpl33*, *rpl36*, *rpoA*, *rpoB*, *rps11*, *rps12*, *rps14*, *rps18*, *rps19*, *rps2*, *rps3*, *rps7*, *rps8*, *ycf1*, and *ycf2*) were extracted from the Cp genomes of eight Rhizophoraceae species and 47 other Malpighiales species (NCBI accession numbers of these Cp genomes were listed in Table S1) and aligned using MAFFT software (Katoh & Standley, 2013). An ML phylogenetic tree was constructed using MEGA X (Kumar et al., 2018), with 10,000 bootstrap replicates. A GTR+T+G nucleotide model was selected as the optimal substitution model. BEAST 2 was employed for estimation of divergence time of Rhizophoraceae species in Malpighiales. Based on the fossil records of *Rhizophora* (∼45 million years ago, Mya) and *Bruguiera* (∼50 Mya) (Graham, 2006), the lognormal prior distribution of Rhizophoraceae was set with an offset value of 45 Mya, a mean of 2 Mya, and a standard deviation of 0.5 Mya (Bouckaert et al., 2014). The chain length for MCMC was set as 200 million generations and 10% for pre-burn-in. FigTree v.1.4.2 was used to draw the phylogenetic tree with annotations (http://tree.bio.ed.ac.uk/software/figtree/).

The phylogenetic relationships among Rhizophoraceae species were then inferred using the LSC regions of the eight investigated Cp genomes. The phylogenetic tree was constructed following the same procedure described above. The divergence among seven Rhizophoraceae species, except F1-hybrid species *R.* × *lamarckii*, was estimated using BEAST 2. The topological structure of the tree prior was set according to the constructed MP tree, and a calibration point was set at the root node of all the seven Rhizophoraceae species according to the estimated divergence time in Malpighiales, where the offset was set at 58.54 Mya and sigma was set as 0.5.

## Results

### Basic characteristics of the chloroplast genomes of four Rhizophoraceae mangrove species

**Figure 1:**
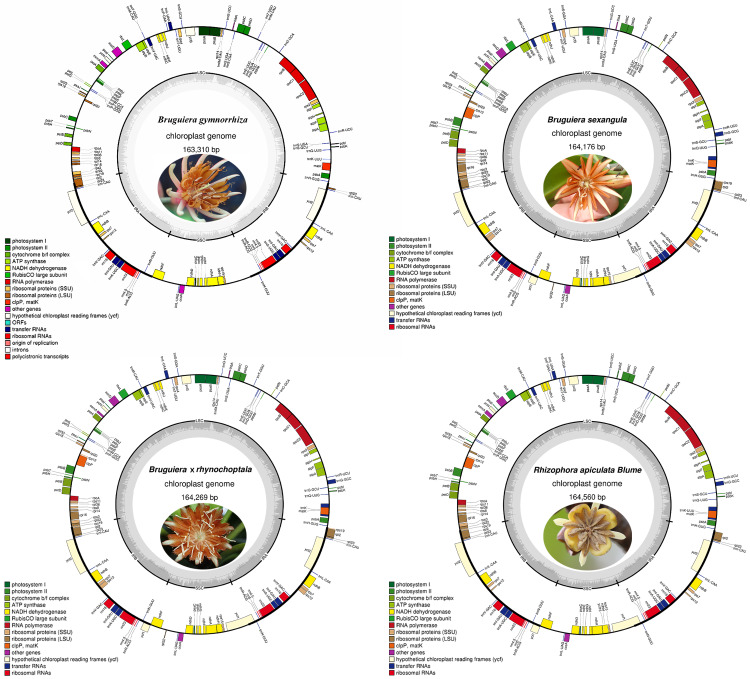
Gene maps of four novel Rhizophoraceae mangrove chloroplast genomes. Genes shown outside the outer circle are transcribed clockwise, and those inside are transcribed counterclockwise. Genes belonging to different functional groups are color-coded. The gray inner circles indicate the GC contents of the chloroplast genomes.

The lengths of the whole Cp genomes are 163,310, 164,269, 164,176, and 164,560 bp for *B. gymnorrhiza*, *B. sexangula*, *B.* × *rhynchopetala* and *R. apiculate*, with conserved structures consisting of a pair of IRs (25,409 bp, 26,393 bp, 36,379 bp and 26,344 bp) that were separated by LSC (92,321 bp, 91,332 bp, 91,304 bp and 93,517 bp) and SSC (20,171 bp, 20,058 bp, 20,207 bp and 19,355 bp) regions (Fig. 1). Conserved characteristics were observed in eight Rhizophoraceae mangrove species (Table 1). Functional genes were identified in all four species, including 80, 81, 81 and 80 protein-coding genes; 38, 39, 37 and 37 tRNA genes; and eight rRNA genes (Table S2).

### Comparative chloroplast genome analyses of Rhizophoraceae mangroves

Protein-coding genes of all eight Rhizophoraceae mangrove species were highly similar with the number of genes ranging from 14 to 19 in IRs. Deletion events in the Cp genomes and various intron containing genes were observed in various Rhizosphoraceae mangrove species (Table 2).

**Figure 2:**
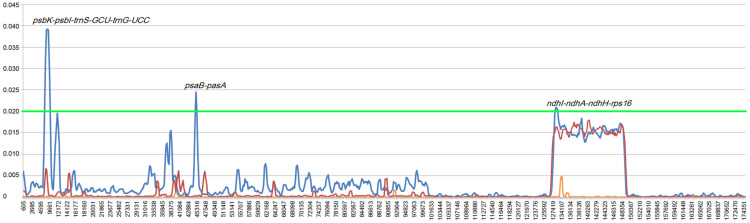
Nucleotide diversity of Rhizophoraceae mangrove chloroplast genomes. The *x*-axis represents the base sequence of the alignment, and the *y*-axis represents Pi values of corresponding sites. The blue line indicates the Pi of eight Rhizophoraceae genomes; the orange line indicates the Pi of three *Bruguiera* chloroplast genomes; the red line indicates the Pi of three *Rhizophora* chloroplast genomes; and the green line indicates the threshold for Pi = 0.02.

**Figure 3:**
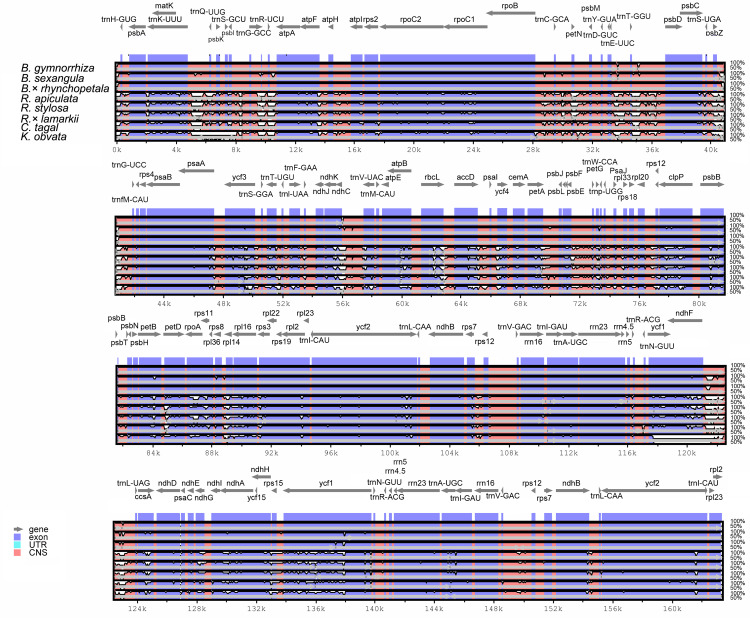
Visualization of the alignment of chloroplast genome sequences of Rhizophoraceae mangrove species. The graphical information from VISTA-based similarity showed the sequence identity of chloroplast genomes of the subtribe Rhizophoreae with *B. gymnorrhiza* as a reference. Gray arrows above the alignment indicate the orientation of genes. Purple bars represent exons, and pink bars represent noncoding sequences. A cutoff of 50% identity was used for the plots. The *Y*-scale axis represents the percent identity from 50 to 100%.

**Figure 4:**
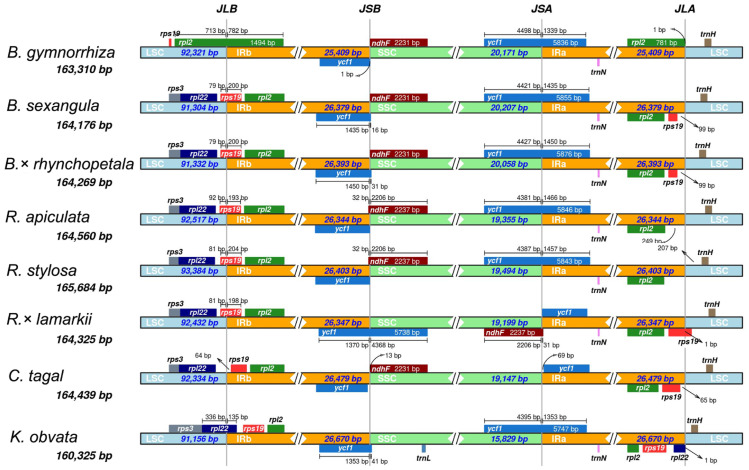
IR contraction/expansion analysis of eight Rhizophoraceae mangrove species. JLB (LSC/IRb), JSB (IRb/SSC), JSA (SSC/IRa) and JLA (IRa/LSC) denote the junction sites between each corresponding region of the genome.

To estimate genetic variation among Rhizophoraceae Cp genomes, nucleotide diversity (Pi) values were calculated using DnaSP software. The average Pi value for all eight Rhizophoraceae Cp genomes was 0.00596, while the average Pi value for the *Bruguiera* and *Rhizophora* Cp genomes was 0.00111 and 0.00472, respectively. The IRs showed lower nucleotide diversity than the single-copy regions, with the highest Pi value observed in the *psbK-psbI-trnS-GCU-trnG-UCC* region (Pi = 0.03898) (Fig. 2). Pairwise alignment among the eight Cp genomes showed high synteny, with relatively lower identity observed in intergenic regions (Fig. 3). The IR boundaries of the eight Cp genomes were also compared, with variations observed among these species (Fig. 4).

### SSRs, codon usage and putative RNA editing sites in chloroplast genes of Rhizophoraceae species

**Figure 5:**
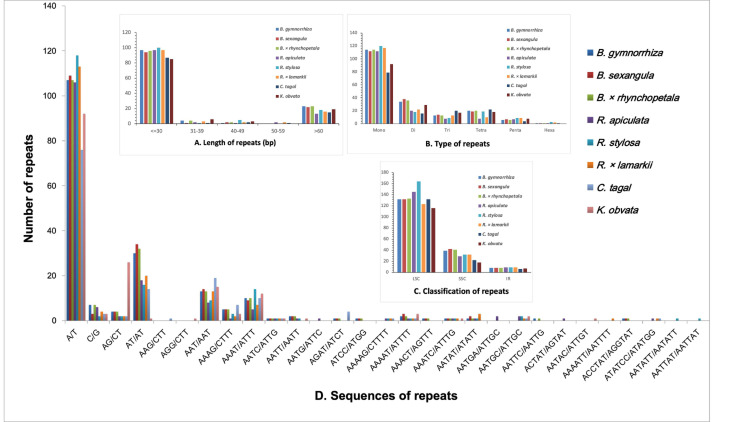
The type and distribution of repeated sequences and SSRs in the chloroplast genomes of eight Rhizophoraceae mangrove species. (A) Repeat sequence length distribution; (B) numbers of six SSR types; (C) numbers of repeat sequences in the LSC, SSC and IR regions; and (D) numbers of identified SSR motifs in different repeat class types.

SSRs (Zalapa et al., 2012) were detected in all eight Rhizophoraceae Cp genomes, with 142 to 191 SSRs identified (Table S3). Mononucleotide and dinucleotide repeats were the most abundant SSRs (Fig. 5). SSRs were mostly located in the LSC regions rather than in the IR and SSC regions, which is consistent with observation in other higher plants (Yang et al., 2019). Some types of SSRs were unique to a particular species or genus (Table S4).

**Figure 6:**
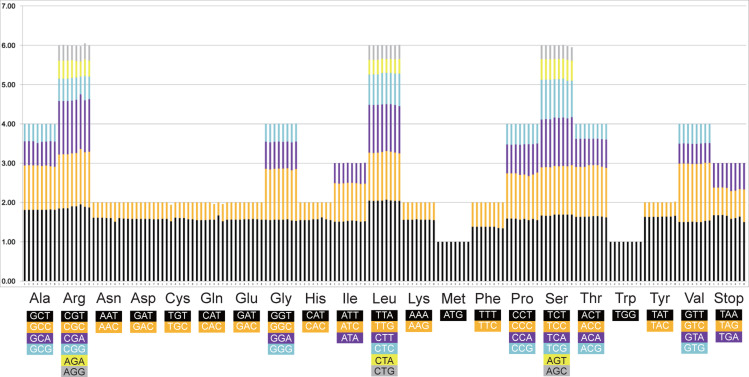
Twenty amino acid codons and stop codons in the chloroplast genomes of eight Rhizophoraceae mangrove species. The colors of the columns correspond to the colors of the codon beneath each amino acid symbol.

The codon numbers in the eight Rhizophoraceae Cp genomes ranged from 21,956 (in *K. obovata*) to 22,822 (in *B. gymnorrhiza*). The start codon ATG was used by most of protein-coding genes, with the remaining genes using ATT (*ycf1*, *rpoA*, *psbB*, *ccsA*, *cemA*, *ndhK* and *rpoC2*), ATA (*ndhA* and *rpoB*), AAA (*psbA* and *cemA*), GTG (*psbC* and *ycf4*), TTG (*rps19* and *ycf4*), ACG (*accD*) and ATC (*ycf2* and *rps2*). The most common stop codon was TAA, followed by TAG and TGA. The relative synonymous codon usage (RSCU) values of the eight Rhizophoraceae mangrove species are shown in Fig. 6, with leucine using the most frequently used codon TTA, followed by serine (TCT) and arginine (GGT).

**Figure 7:**
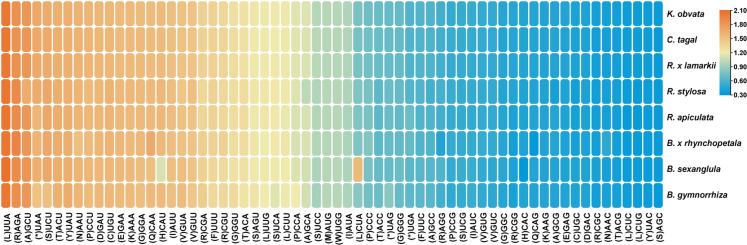
Codon usage in the form of a heatmap for eight Rhizophoraceae mangrove species. Color indication: pink represents higher RSCU values, and blue represents lower RSCU values.

The distribution of codon usage in the form of heatmaps for the eight Rhizophoraceae mangrove species was shown (Fig. 7). Codons with RSCU >1 ended with A/U, except for UUG (L), which is consistent with similar findings in other plant lineages (Zhang et al., 2019). This result supported the theory that A+T (U) bias plays an important role in the plant Cp genome with the reduction in GC content. Moreover, the codon usage of eight Rhizophoraceae mangrove species was evolutionarily conserved, with three *Bruguiera* species and three *Rhizophora* species clustered into separate groups (Fig. 7).

**Table 1:** Comparison of the basic characteristics of the chloroplast genomes of eight Rhizophoraceae species.

**Genus**	***Bruguiera***			***Rhizophora***			***Ceriops***	***Kandelia***
**Species**	B. gymnorrhiza^*^	B. sexangular^*^	***B.*×** rhynchopetala^*^	R. apiculate^*^	***R. stylosa***	***R.*×* lamarkii***	***C. tagal***	***K. obvata***
Length (bp)	163,310	164,269	164,176	164,560	165,684	164,325	164,439	160,325
GC content (%)	35.2	35.2	35.2	34.9	34.9	34.7	35.4	35.2
LSC length (bp)	92,321	91,332	91,304	93,517	93,384	92,432	92,334	91,156
GC content (%)	32.9	32.8	32.8	32.1	32.0	32.1	32.7	29.1
SSC length (bp)	20,171	20,058	20,207	19,355	19,494	19,199	19,147	15,829
GC content (%)	28.1	28.0	28.0	28.5	28.6	28.6	29.2	32.3
IR length (bp)	25,409	26,393	26,379	26,344	26,403	26,347	26,479	26,670
GC content (%)	40.3	42.1	42.1	42.2	42.2	42.2	42.1	42.0
Gene number	126	128	126	125	124	126	127	126
Gene number in IR regions	15	17	16	14	14	16	16	19
Protein-coding gene number	80	81	81	80	79	81	81	80
Protein-coding gene (%)	63.5	63.2	64.3	64.0	63.7	64.3	63.8	63.5
rRNA gene number	8	8	8	8	8	8	8	8
rRNA (%)	6.4	6.3	6.3	6.4	6.4	6.3	6.3	6.4
tRNA gene number	38	39	37	37	37	37	38	38
tRNA (%)	30.2	30.5	29.4	29.6	29.8	29.4	29.9	30.2

**Notes.**

* represents the four species reported in this study.

**Table 2:** Intron-containing genes in the chloroplast genomes of Rhizophoraceae mangroves.

Species	Genes contain one inton	Genes contain two introns
*B. gymnorrhiza*	*rpoC1, atpF, ndhA, ndhB, ycf1 , trnV-UAC, trnL-UAA, trnK-UUU , trnI-GAU, trnG-UCC ,trnA-UGC*	*rps12, clpP, ycf3*
*B. sexangula*	*rps19, rpoC1, atpF, ndhA, ndhB, ycf1 , trnV-UAC, trnL-UAA, trnK-UUU , trnI-GAU, trnA-UGC*	*rps12, clpP, ycf3*
*B.*×* rhynchopetala*	*rps19, rpoC1, atpF, ndhA, ndhB, ycf1 , trnV-UAC, trnL-UAA, trnK-UUU , trnI-GAU, trnA-UGC*	*rps12, clpP, ycf3*
*R. apiculata*	*rpl16, rpoC1, atpF, ndhA, ndhB,petB , petD , ycf1 , trnV-UAC, trnL-UAA, trnK-UUU , trnI-GAU, trnG-GCC,trnA-UGC*	*rps12, clpP, ycf3*
*R. stylosa*	*rpl16, rpoC1, atpF, ndhA, ndhB, petB , petD, ycf1, trnV-UAC, trnL-UAA, trnK-UUU , trnI-GAU, trnA-UGC*	*rps12, clpP, ycf3*
*R.*×* lamarkii*	*rps19, rpl2, rpl16, rpoC1, atpF, ndhA, ndhB, petB , petD , ycf1 , trnV-UAC, trnL-UAA, trnK-UUU , trnI-GAU, trnA-UGC*	*rps12, clpP, ycf3*
*C. tagal*	*rpl2, rpl22, rpoC1, atpF, ndhA, ndhB, petB , petD*, *trnV-UAC, trnL-UAA,trnI-GAU, trnA-UGC*	*rps19, rps12, clpP,ycf1*, *ycf3*
*K. obvata*	*rpl2, rpoC1, atpF, ndhA, ndhB, petB , petD , ycf1 , trnV-UAC, trnL-UAA, trnK-UUU,trnI-GAU, trnA-UGC*	*rps12, clpP, ycf3*

Predicted RNA editing sites were compared in the Cp genomes of eight Rhizophoraceae mangrove species, revealing that *R. stylosa* has the most RNA editing sites, followed by *B. gymnorrhiza*, *B. sexangula*, *B.* × *rhynchopetala*, *R. apiculata* and *R. × lamarckii* (Table 3 and Table S5). Some RNA editing sites were unique to a particular genus or species, such as T =>I in *accD* only in the *Bruguiera* genus and A =>V in *atpB* and T =>I in *rpoC1* only in *Rhizophora* (Table S5). Additionally, some RNA editing sites were found in *K. obovata* and/or *C. tagal* but not in *Bruguiera* and *Rhizophora* species, such as R =>W in *rpoC2*, S =>L in *rpoA*, P =>L in *matK* and H =>Y in *ndhA* in *K. obovata* and S = >F in *ndhA* and S = >L in *rpoC2* in *C. tagal*. Forty-two editing sites distributed in 16 genes were shared across all eight species and showed higher conservation than that in some other plants.

### Divergence time of Rhizophoraceae species

**Figure 8:**
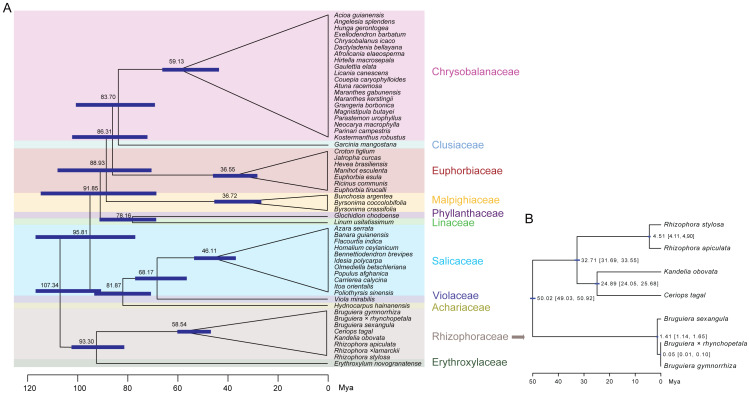
Divergence time of Rhizophoraceae mangrove species. (A) Divergence time of Rhizophoraceae species in Malpighiales. (B) Divergence time of *Rhizophora*, *Kandelia*, *Ceriops* and *Bruguiera* species within Rhizophoraceae. 95% confidence intervals (CI) are listed in the brackets.

Divergence time estimation in Malpighiales showed that Rhizophoraceae and Erythroxylaceae formed a group and diverged approximately ∼93.30 Mya (Fig. 8A). The basal branch between this group and other Malpighiales species diverged approximately ∼107.34 Mya. The Rhizophoraceae mangrove species diverged from each other about ∼58.54 Mya. Phylogenetic analysis in Rhizophoraceae revealed that *C. tagal* formed a group with *K. obovata*, and Rhizophora species were sister to *C. tagal* and *K. obovata* (Fig. S1). It is worthy to note that, hybrid species *R.* ×* lamarckii* fell into the same group with its female parent *R. stylosa*, which is consistent to our expectation. Comparatively, *Bruguiera* species showed the largest divergence to other Rhizophoraceae species. The divergence time estimation suggested that the genus *Bruguiera* separated from the other species at approximately 50.02 Mya (95% CI [49.03–50.92] Mya), and the *Rhizophora* species diverged from *C. tagal* and *K. obovata* at ∼32.71 Mya (95% CI [31.69–33.55] Mya) (Fig. 8B). The divergence between *C. tagal* and *K. obovata* was supposed to occur at 24.89 Mya and the split time of *R. apiculate* and *R. stylosa* was ∼4.51 Mya (Fig. 8B).

## Discussion

### The structural characteristics of the chloroplast genomes of Rhizophoraceae mangroves

The chloroplast genomes of higher plants are highly conserved in terms of genome structure, gene order, and gene content (Daniell et al., 2016). In this study, we found that the same typical structure also exists in the four newly reported Cp genomes of Rhizophoraceae mangrove species. Their genome sizes are dynamic and primarily influenced by transposon and large fragment indel events, consisting with previous report (Chen et al., 2017). The intertidal habitat of mangroves is characterized by high salinity, hypoxia, and many other abiotic stresses, which may accelerate DNA transfer from chloroplasts and mitochondria to the nucleus (Wang & Timmis, 2013; Feng et al., 2021). However, despite these environmental stresses, the Cp genome sizes of Rhizophoraceae mangroves are among the largest reported to date in Malpighiales, ranging from 160,325 bp to 165,684 bp (Bedoya et al., 2019).

**Table 3:** RNA editing sites shared by the eight chloroplast genomes of Rhizophoraceae mangroves predicted by PREP.

Gene	A.A position	*B. gymnorrhiza*	*B. sexangula*	*B. × rhynchopetala*	*R. apiculata*	*R. stylosa*	*R. × lamarkii*	*C. tagal*	*K. obvata*
		Codon(A.A) conversion							
*accD*	269	TCG (S) =>TTG (L)	TCG (S) =>TTG (L)	TCG (S) =>TTG (L)	TCG (S) =>TTG (L)	TCG (S) =>TTG (L)	TCG (S) =>TTG (L)	TCG (S) =>TTG (L)	TCG (S) =>TTG (L)
	472	CCT (P) =>CTT (L)	CCT (P) =>CTT (L)	CCT (P) =>CTT (L)	CCT (P) =>CTT (L)	CCT (P) =>CTT (L)	CCT (P) =>CTT (L)	CCT (P) =>CTT (L)	CCT (P) =>CTT (L)
*atpF*	31	CCA (P) =>CTA (L)	CCA (P) =>CTA (L)	CCA (P) =>CTA (L)	CCA (P) =>CTA (L)	CCA (P) =>CTA (L)	CCA (P) =>CTA (L)	CCA (P) =>CTA (L)	CCA (P) =>CTA (L)
*atpI*	210	TCA (S) =>TTA (L)	TCA (S) =>TTA (L)	TCA (S) =>TTA (L)	TCA (S) =>TTA (L)	TCA (S) =>TTA (L)	TCA (S) =>TTA (L)	TCA (S) =>TTA (L)	TCA (S) =>TTA (L)
*clpP*	187	CAT (H) =>TAT (Y)	CAT (H) =>TAT (Y)	CAT (H) =>TAT (Y)	CAT (H) =>TAT (Y)	CAT (H) =>TAT (Y)	CAT (H) =>TAT (Y)	CAT (H) =>TAT (Y)	CAT (H) =>TAT (Y)
*matK*	178	CTC (L) =>TTC (F)	CTC (L) =>TTC (F)	CTC (L) =>TTC (F)	CTC (L) =>TTC (F)	CTC (L) =>TTC (F)	CTC (L) =>TTC (F)	CTC (L) =>TTC (F)	CTC (L) =>TTC (F)
	392	CGG (R) =>TGG (W)	CGG (R) =>TGG (W)	CGG (R) =>TGG (W)	CGG (R) =>TGG (W)	CGG (R) =>TGG (W)	CGG (R) =>TGG (W)	CGG (R) =>TGG (W)	CGG (R) =>TGG (W)
	396	TCA (S) =>TTA (L)	TCA (S) =>TTA (L)	TCA (S) =>TTA (L)	TCA (S) =>TTA (L)	TCA (S) =>TTA (L)	TCA (S) =>TTA (L)	TCA (S) =>TTA (L)	TCA (S) =>TTA (L)
*ndhB*	50	TCA (S) =>TTA (L)	TCA (S) =>TTA (L)	TCA (S) =>TTA (L)	TCA (S) =>TTA (L)	TCA (S) =>TTA (L)	TCA (S) =>TTA (L)	TCA (S) =>TTA (L)	TCA (S) =>TTA (L)
	156	CCA (P) =>CTA (L)	CCA (P) =>CTA (L)	CCA (P) =>CTA (L)	CCA (P) =>CTA (L)	CCA (P) =>CTA (L)	CCA (P) =>CTA (L)	CCA (P) =>CTA (L)	CCA (P) =>CTA (L)
	196	CAT (H) =>TAT (Y)	CAT (H) =>TAT (Y)	CAT (H) =>TAT (Y)	CAT (H) =>TAT (Y)	CAT (H) =>TAT (Y)	CAT (H) =>TAT (Y)	CAT (H) =>TAT (Y)	CAT (H) =>TAT (Y)
	204	TCG (S) =>TTG (L)	TCG (S) =>TTG (L)	TCG (S) =>TTG (L)	TCG (S) =>TTG (L)	TCG (S) =>TTG (L)	TCG (S) =>TTG (L)	TCG (S) =>TTG (L)	TCG (S) =>TTG (L)
	246	CCA (P) =>CTA (L)	CCA (P) =>CTA (L)	CCA (P) =>CTA (L)	CCA (P) =>CTA (L)	CCA (P) =>CTA (L)	CCA (P) =>CTA (L)	CCA (P) =>CTA (L)	CCA (P) =>CTA (L)
	249	TCT (S) =>TTT (F)	TCT (S) =>TTT (F)	TCT (S) =>TTT (F)	TCT (S) =>TTT (F)	TCT (S) =>TTT (F)	TCT (S) =>TTT (F)	TCT (S) =>TTT (F)	TCT (S) =>TTT (F)
	277	TCA (S) =>TTA (L)	TCA (S) =>TTA (L)	TCA (S) =>TTA (L)	TCA (S) =>TTA (L)	TCA (S) =>TTA (L)	TCA (S) =>TTA (L)	TCA (S) =>TTA (L)	TCA (S) =>TTA (L)
	279	TCA (S) =>TTA (L)	TCA (S) =>TTA (L)	TCA (S) =>TTA (L)	TCA (S) =>TTA (L)	TCA (S) =>TTA (L)	TCA (S) =>TTA (L)	TCA (S) =>TTA (L)	TCA (S) =>TTA (L)
	419	CAT (H) =>TAT (Y)	CAT (H) =>TAT (Y)	CAT (H) =>TAT (Y)	CAT (H) =>TAT (Y)	CAT (H) =>TAT (Y)	CAT (H) =>TAT (Y)	CAT (H) =>TAT (Y)	CAT (H) =>TAT (Y)
	494	CCA (P) =>CTA (L)	CCA (P) =>CTA (L)	CCA (P) =>CTA (L)	CCA (P) =>CTA (L)	CCA (P) =>CTA (L)	CCA (P) =>CTA (L)	CCA (P) =>CTA (L)	CCA (P) =>CTA (L)
*ndhD*	182	GCT (A) =>GTT (V)	GCT (A) =>GTT (V)	GCT (A) =>GTT (V)	GCT (A) =>GTT (V)	GCT (A) =>GTT (V)	GCT (A) =>GTT (V)	GCT (A) =>GTT (V)	GCT (A) =>GTT (V)
	293	TCA (S) =>TTA (L)	TCA (S) =>TTA (L)	TCA (S) =>TTA (L)	TCA (S) =>TTA (L)	TCA (S) =>TTA (L)	TCA (S) =>TTA (L)	TCA (S) =>TTA (L)	TCA (S) =>TTA (L)
	359	GCT (A) =>GTT (V)	GCT (A) =>GTT (V)	GCT (A) =>GTT (V)	GCT (A) =>GTT (V)	GCT (A) =>GTT (V)	GCT (A) =>GTT (V)	GCT (A) =>GTT (V)	GCT (A) =>GTT (V)
	369	ACC (T) =>ATC (I)	ACC (T) =>ATC (I)	ACC (T) =>ATC (I)	ACC (T) =>ATC (I)	ACC (T) =>ATC (I)	ACC (T) =>ATC (I)	ACC (T) =>ATC (I)	ACC (T) =>ATC (I)
	469	CTT (L) =>TTT (F)	CTT (L) =>TTT (F)	CTT (L) =>TTT (F)	CTT (L) =>TTT (F)	CTT (L) =>TTT (F)	CTT (L) =>TTT (F)	CTT (L) =>TTT (F)	CTT (L) =>TTT (F)
*ndhF*	87	CAC (H) =>TAC (Y)	CAC (H) =>TAC (Y)	CAC (H) =>TAC (Y)	CAC (H) =>TAC (Y)	CAC (H) =>TAC (Y)	CAC (H) =>TAC (Y)	CAC (H) =>TAC (Y)	CAC (H) =>TAC (Y)
	97	TCA (S) =>TTA (L)	TCA (S) =>TTA (L)	TCA (S) =>TTA (L)	TCA (S) =>TTA (L)	TCA (S) =>TTA (L)	TCA (S) =>TTA (L)	TCA (S) =>TTA (L)	TCA (S) =>TTA (L)
	105	ACT (T) =>ATT (I)	ACT (T) =>ATT (I)	ACT (T) =>ATT (I)	ACT (T) =>ATT (I)	ACT (T) =>ATT (I)	ACT (T) =>ATT (I)	ACT (T) =>ATT (I)	ACT (T) =>ATT (I)
	196	CTT (L) =>TTT (F)	CTT (L) =>TTT (F)	CTT (L) =>TTT (F)	CTT (L) =>TTT (F)	CTT (L) =>TTT (F)	CTT (L) =>TTT (F)	CTT (L) =>TTT (F)	CTT (L) =>TTT (F)
	478	CCA (P) =>CTA (L)	CCA (P) =>CTA (L)	CCA (P) =>CTA (L)	CCA (P) =>CTA (L)	CCA (P) =>CTA (L)	CCA (P) =>CTA (L)	CCA (P) =>CTA (L)	CCA (P) =>CTA (L)
	643	CTT (L) =>TTT (F)	CTT (L) =>TTT (F)	CTT (L) =>TTT (F)	CTT (L) =>TTT (F)	CTT (L) =>TTT (F)	CTT (L) =>TTT (F)	CTT (L) =>TTT (F)	CTT (L) =>TTT (F)
	709	CTT (L) =>TTT (F)	CTT (L) =>TTT (F)	CTT (L) =>TTT (F)	CTT (L) =>TTT (F)	CTT (L) =>TTT (F)	CTT (L) =>TTT (F)	CTT (L) =>TTT (F)	CTT (L) =>TTT (F)
*ndhG*	105	ACA (T) =>ATA (I)	ACA (T) =>ATA (I)	ACA (T) =>ATA (I)	ACA (T) =>ATA (I)	ACA (T) =>ATA (I)	ACA (T) =>ATA (I)	ACA (T) =>ATA (I)	ACA (T) =>ATA (I)
*petB*	204	CCA (P) =>CTA (L)	CCA (P) =>CTA (L)	CCA (P) =>CTA (L)	CCA (P) =>CTA (L)	CCA (P) =>CTA (L)	CCA (P) =>CTA (L)	CCA (P) =>CTA (L)	CCA (P) =>CTA (L)
*psaI*	28	TCT (S) =>TTT (F)	TCT (S) =>TTT (F)	TCT (S) =>TTT (F)	TCT (S) =>TTT (F)	TCT (S) =>TTT (F)	TCT (S) =>TTT (F)	TCT (S) =>TTT (F)	TCT (S) =>TTT (F)
*rpl2*	199	GCT (A) =>GTT (V)	GCT (A) =>GTT (V)	GCT (A) =>GTT (V)	GCT (A) =>GTT (V)	GCT (A) =>GTT (V)	GCT (A) =>GTT (V)	GCT (A) =>GTT (V)	GCT (A) =>GTT (V)
*rpoA*	296	TCA (S) =>TTA (L)	TCT (S) =>TTT (F)	TCT (S) =>TTT (F)	TCA (S) =>TTA (L)	TCA (S) =>TTA (L)	TCA (S) =>TTA (L)	TCA (S) =>TTA (L)	TCA (S) =>TTA (L)
*rpoB*	113	TCT (S) =>TTT (F)	TCT (S) =>TTT (F)	TCT (S) =>TTT (F)	TCT (S) =>TTT (F)	TCT (S) =>TTT (F)	TCT (S) =>TTT (F)	TCA (S) =>TTA (L)	TCT (S) =>TTT (F)
*rpoC2*	484	CGC (R) =>TGC (C)	CGC (R) =>TGC (C)	CGC (R) =>TGC (C)	CGC (R) =>TGC (C)	CGC (R) =>TGC (C)	CGC (R) =>TGC (C)	CGC (R) =>TGC (C)	CGC (R) =>TGC (C)
	535	CCT (P) =>TTT (F)	CCT (P) =>TTT (F)	CCT (P) =>TTT (F)	CCT (P) =>TTT (F)	CCT (P) =>TTT (F)	CCT (P) =>TTT (F)	CCT (P) =>TTT (F)	CCT (P) =>TTT (F)
	535	CCT (P) =>TTT (F)	CCT (P) =>TTT (F)	CCT (P) =>TTT (F)	CCT (P) =>TTT (F)	CCT (P) =>TTT (F)	CCT (P) =>TTT (F)	CCT (P) =>TTT (F)	CCT (P) =>TTT (F)
	768	GCC (A) =>GTC (V)	GCC (A) =>GTC (V)	GCC (A) =>GTC (V)	GCC (A) =>GTC (V)	GCC (A) =>GTC (V)	GCC (A) =>GTC (V)	GCC (A) =>GTC (V)	GCC (A) =>GTC (V)
*rps14*	27	TCA (S) =>TTA (L)	TCA (S) =>TTA (L)	TCA (S) =>TTA (L)	TCA (S) =>TTA (L)	TCA (S) =>TTA (L)	TCA (S) =>TTA (L)	TCA (S) =>TTA (L)	TCA (S) =>TTA (L)
	50	CCA (P) =>CTA (L)	CCA (P) =>CTA (L)	CCA (P) =>CTA (L)	CCA (P) =>CTA (L)	CCA (P) =>CTA (L)	CCA (P) =>CTA (L)	CCA (P) =>CTA (L)	CCA (P) =>CTA (L)

The Cp genomes of eight Rhizophoraceae mangroves have highly conserved gene structure and characteristics (Table 1, Fig. 1), although some differences were observed in these Cp genomes, such as gene deletion and inversion due to occasional rearrangements (Fig. 4, Table S2). Previous studies have found numerous Cp genome rearrangements in angiosperms, including *Cryptomeria*, *Agathis*, *Nageia* and *Calocedrus* (Zheng et al., 2016), as well as in gymnosperms, including Cupressophytes and Pinaceae (Hao et al., 2016). In Malpighiales, a rearrangement event in the LSC region was reported in Podostemaceae (Ruhfel et al., 2011). In the eight Rhizophoraceae mangroves studied here, a large inversion containing 14 genes was only observed in *R.* ×* lamarckii*, and a similar event can be found in the Cp genome of *Anthoceros formosae* (Kugita et al., 2003). The reliability of this inversion of such a long fragment occurred in the Cp genome should be further validated, and its potential contribution to this natural hybrid requires further investigation.

The present study identified single-gene divergences in addition to large fragment structure variations in the Rhizophoraceae Cp genomes. For example, the *ndhF* gene was absent from the Cp genome of *K. obovata*, which is consist with previous research (Yang et al., 2019). In plants from the same family, highly diverse regions result from the loss or gain of introns within protein-coding genes (Babenko et al., 2004). The *matK* gene, which encodes the unique maturase in the plastid genomes of land plants, was found in the intronic region of the *trnK-UUU* gene in Passifloraceae (Cauz-Santos et al., 2020), Euphorbiaceae (Wang et al., 2020), and the Rhizophoraceae genomes studied here (Fig. 4). The *clpP* gene with two introns in Rhizophoraceae was found to have no introns or one intron in other Malpighiales species, such as *Populus trichocarpa* (Zhu et al., 2018), *Manihot esculenta* (Daniell et al., 2008), and *Jatropha curcas* (Asif et al., 2010), indicating that the *clpP* gene may be undergoing functional divergence. However, previous research showed that introns were observed in the *atpF* gene in 251 taxa of Malpighiales but were absent in other Malpighiales species and in the closely related family Euphorniaceae (Daniell et al., 2008). Nonetheless, the *atpF* genes in eight Rhizophoraceae mangroves had one intron (Table 2). These structural variations within protein-coding genes in eight Rhizophoraceae mangroves indicate a dynamic evolutionary process.

### Phylogenetic analyses provided insight into the systematic evolutionary relationships and divergence ages of Rhizophoraceae mangrove plants

This work provides a comprehensive evolutionary analysis based on whole-genome Cp sequences of all eight Rhizophoraceae mangrove species distributed in China. Within Malpighiales, the phylogenetic relationships found here are in line with previous work that claimed Salicaceae and Violaceae share a most recent common ancestor (Davis et al., 2005). The origin of Rhizophoraceae was dated at ∼58.54–50.02 Mya (Fig. 8), which is similar to the estimation using whole-genome sequencing data (∼54.6–47.8 Mya) (Xu et al., 2017b). In the period, due to the markable Paleocene-Eocene Thermal Maximum, annual air temperatures raised by 5–10 °C and sea levels changed dramatically (Naafs et al., 2018), which were supposed to offer the opportunities for mangrove plants to enter the intertidal regions.

Morphology studies divided Rhizophoraceae into four subgroups (van Vliet, 1976b). However, the APG IV system, a well-known taxonomical system based mainly on chloroplast sequences, supports a three-cluster classification for Rhizophoraceae: Macarisieae, Gynotrocheae, and Rhizophoreae (The Angiosperm Phylogeny Group et al., 2016). In this study, our results revealed clear phylogenetic relationships among the Rhizophoreae species: *Rhizophora* species were sister to the cluster of *C. tagal* and *K. obovata*, while *Bruguiera* species were less closely related to these species (Fig. 8B; Fig. S1). The divergence time of *C. tagal* and *K. obovata* was estimated to be ∼24.89 Mya and the split between *Rhizophora* and the cluster of *C. tagal* and *K. obovata* might occur at ∼32.71 Mya (Fig. 8B), which were also agreed with those from whole-genome sequencing results (Xu et al., 2017b). We further inferred the divergence time of *R. apiculate* and *R. stylosa* to be ∼4.51 Mya, suggesting a recent speciation process in *Rhizophora*. These estimations were more accurate than previous inferences using few Cp or nuclear loci (Chen et al., 2015; Menezes et al., 2018; Schwarzbach & Ricklefs, 2000), indicating the powerfulness of using multi-locus sequences for phylogenetic relationship inference and divergence time dating of close-related species (Yang et al., 2015). Cp sequences in Rhizophoraceae mangrove species also provide an alternative means for elucidating the direction of hybridization and introgression at the species level and shed light on the origin and evolution of the mangrove hybrid, which was previously unclear based on morphological characters alone (Lo, Duke & Sun, 2014). Hybrid species *B. × rhynchopetala* was more closely related to *B. gymnorrhiza* than *B. sexangular* (Fig. 8B), suggesting that *B. gymnorrhiza* was the female parent of the *B. × rhynchopetala* individual we collected in this study. However, in other scenarios, the hybridization can occur in the reverse direction as well. Thus, more genomic information is necessary for a comprehensive understanding of the divergence and inter-species gene flow among *Bruguiera* mangrove species.

## Conclusions

We have successfully assembled and analyzed the complete Cp genomes of four Rhizophoraceae mangroves, finding that the gene contents and orders were highly conserved. Notably, we identified three regions with Pi values greater than 0.02: *psbK-psbI-trnS-GCU-trnG-UCC*, *psaB-pasA* and *ndhI-ndhA-ndhH-rps16*. In addition, we observed a large inversion containing 14 genes that was unique to *R. × lamarckii*. Phylogenetic and divergence time estimation based on the Cp genomes of Malpighiales revealed that the origin of Rhizophoraceae mangrove species occurred at around 58.54–50.02 Mya, and *R. apiculate* and *R. stylosa* were diverged at ∼4.51 Mya. Our study provides comprehensive evolutionary analyses of the Cp genomes of all eight Rhizophoraceae mangrove species in China. Our findings shed light on the origin and evolution of mangrove hybrids. However, more genomic information and fossil records are needed to determine the exact evolutionary history of different genera among Rhizophoraceae mangrove species.

##  Supplemental Information

10.7717/peerj.16400/supp-1 Supplemental Information 1 Supplemental Figure and Tables

## Additional Information and Declarations

### DNA Deposition

The following information was supplied regarding the deposition of DNA sequences: The chloroplast genome sequences are available at GenBank: MT129628 to MT129631.

### Data Availability

The following information was supplied regarding data availability: The sequences are available at: NCBI SRA SRR15235729, SRR15235730, SRR15235731, SRR15235732.
