# Supplementary material for: Comparative chloroplast genome analyses provide insights into evolutionary history of Rhizophoraceae mangroves
Source: PeerJ. 2023 Nov 17;11:e16400. doi: 10.7717/peerj.16400 (PMC10658886; doi:10.7717/peerj.16400)
Supplement: Supplemental Information 1 [file peerj-11-16400-s001.docx]

**Comparative chloroplast genome analyses provide insights into evolutionary history of Rhizophoraceae mangroves**

Ying Zhang^1,2,3#^, Yuchen Yang^4#^, Meng He^5^, Ziqi Wei^5^, Xi Qin^5^, Yuanhao Wu^5^, Qingxing Jiang^5^, Yufeng Xiao^5^, Yong Yang^5^, Wei Wang^2*^ & Xiang Jin^2,5,*^

^1^ Hainan Academy of Forestry, Hainan Mangrove Research Institute, Haikou, Hainan, 571100, China

^2^ Research Center for Wild Animal and Plant Resource Protection and Utilization, Qiongtai Normal University, Haikou, Hainan, 571127, China

^3^ Life Science and Technology School, Lingnan Normal University, Zhanjiang, Guangdong, 524048, China

^4^ State Key Laboratory of Biocontrol, School of Ecology, Sun Yat-sen University, Shenzhen 518107, China

^5^ Ministry of Education Key Laboratory for Ecology of Tropical Islands, Key Laboratory of Tropical Animal and Plant Ecology of Hainan Province, College of Life Sciences, Hainan Normal University, Haikou, Hainan, 571158, China

Corresponding Authors:

Xiang Jin^2,4^

99# Longkunnan Road, Hainan Normal University, Haikou, Hainan, 571158, China

Email address: jinx@hainnu.edu.cn

Wei Wang^2^

Qiongtai Normal University, Research center for wild animal and plant resource protection and utilization, Haikou, Hainan, China

Email address: wwinsect@mail.qtnu.edu.cn

^#^ These authors contributed equally to this work.

**Table S1.** NCBI accession numbers of the Cp genomes of 47 Malpighiales species used for phylogenetic analysis

| Species | Accession number |
| --- | --- |
| *Acioa guianensis* | NC_030534 |
| *Angelesia splendens* | NC_030545 |
| *Hunga gerontogea* | NC_030564 |
| *Exellodendron barbatum* | NC_030558 |
| *Chrysobalanus icaco* | NC_024061 |
| *Dactyladenia bellayana* | NC_030555 |
| *Afrolicania elaeosperma* | NC_030544 |
| *Hirtella macrosepala* | NC_030561 |
| *Gaulettia elata* | NC_030559 |
| *Licania canescens* | NC_030566 |
| *Couepia caryophylloides* | NC_030547 |
| *Atuna racemosa* | NC_030546 |
| *Maranthes gabunensis* | NC_030577 |
| *Maranthes kerstingii* | NC_030579 |
| *Grangeria borbonica* | NC_030560 |
| *Magnistipula butayei* | NC_030576 |
| *Parastemon urophyllus* | NC_030517 |
| *Neocarya macrophylla* | NC_030580 |
| *Parinari campestris* | NC_024067 |
| *Kostermanthus robustus* | NC_030565 |
| *Garcinia mangostana* | NC_036341 |
| *Croton tiglium* | NC_040113 |
| *Jatropha curcas* | NC_012224 |
| *Hevea brasiliensis* | NC_015308 |
| *Manihot esculenta* | NC_010433 |
| *Euphorbia esula* | NC_033910 |
| *Ricinus communis* | NC_016736 |
| *Euphorbia tirucalli* | NC_042193 |
| *Bunchosia argentea* | NC_041491 |
| *Byrsonima coccolobifolia* | NC_037191 |
| *Byrsonima crassifolia* | NC_037192 |
| *Glochidion chodoense* | NC_042906 |
| *Linum usitatissimum* | NC_036356 |
| *Azara serrata* | NC_041433 |
| *Banara guianensis* | NC_043896 |
| *Flacourtia indica* | NC_037410 |
| *Homalium ceylanicum* | NC_045235 |
| *Bennettiodendron brevipes* | NC_043885 |
| *Idesia polycarpa* | NC_032060 |
| *Olmediella betschleriana* | NC_043886 |
| *Populus afghanica* | NC_045396 |
| *Carrierea calycina* | NC_043884 |
| *Itoa orientalis* | NC_037411 |
| *Poliothyrsis sinensis* | NC_037412 |
| *Viola mirabilis* | NC_041582 |
| *Hydnocarpus hainanensis* | NC_042720 |
| *Bruguiera gymnorrhiza* | MT129628 |
| *Bruguiera × rhynchopetala* | MT129630 |
| *Bruguiera sexangula* | MT129629 |
| *Ceriopes tagal* | MH240830.1 |
| *Kandelia obovata* | NC_042718 |
| *Rhizophora apiculata* | MT129631 |
| *Rhizophora × lamarkii* | MK392466.1 |
| *Rhizophora stylosa* | NC_042819 |
| *Erythroxylum novogranatense* | NC_030601 |

**Table S2.** Genes of the Cp genome of eight Rhizophoraceae mangroves.

| **Species** | *Bruguiera gymnorrhiza* | |
| --- | --- | --- |
| **Functions Category** | **Group of Genes** | **Gene Name** |
| **Self-replication** | Small subunit of ribosome | *rps2, rps3, rps4, rps7^a^, rps8, rps11,rps12^acde^, rps14, rps15, rps18, rps19* |
|  | large subunit of ribosome | *rpl2^ad^, rpl14, rpl16, rpl20,rpl22, rpl23^a^, rpl32,rpl33, rpl36* |
|  | rRNA genes | *rrn4.5^a^, rrn5^a^, rrn16^a^, rrn23^a^* |
|  | DNA-dependent RNA polymerase | *rpoA, rpoB, rpoC1^b^, rpoC2* |
|  | rRNA Genes | *trnY-GUA, trnW-CCA, trnV-UAC^b^, trnV-GAC^a^, trnT-UGU, trnT-GGU, trnS-UGA, trnS-GGA, trnS-GCU, trnR-UCU, trnR-ACG^a^, trnQ-UUG, trnP-UGG, trnN-GUU^a^,trnM-CAU, trnL-UAG, trnL-UAA^b^, trnL-CAA^a^, trnK-UUU^b^, trnI-GAU^ab^, trnI-CAU^a^, trnH-GUG, trnG-GCC, trnG-UCC^ab^, trnfM-CAU, trnF-GAA, trnE-UUC, trnD-GUC, trnC-GCA, trnA-UGC^ab^* |
| **Genes for Photosynthesis** | Subunits of ATP synthase | *atpA, atpB, atpE, atpF^b^, atpH, atpI* |
|  | Subunits of NADH-dehydrogenase | *ndhA^b^, ndhB^ab^, ndhC, ndhD, ndhE, ndhF, ndhG, ndhH, ndhI, ndhJ, ndhK* |
|  | Subunits of cytochrome b/f comples | *petA, petB, petD, petG, petL, petN* |
|  | Subunits of photosystem I | *psaA, psaB, psaC, psaI, psaJ* |
|  | Subunits of photosystem II | *psbA, psbB, psbC, psbD, psbE, psbF, psbH, psbI, psbJ, psbK, psbL, psbM, psbN, psbT, psbZ* |
|  | Subunits of rubisco | *rbcL* |
| **Other Genes** | Subunits of Acetyl-CoA-carboxylase | *accD* |
|  | Envelop membrane protein | *cemA* |
|  | c-type cytochrome synthesis gene | *ccsA* |
|  | Protease | *clpP^c^* |
|  | Translational initiation factor |  |
|  | Maturase | *matK* |
|  | Elongation factor |  |
| **Genes of Unknown Function** | Conserved open reading frames | *ycf1^abd^, ycf2^a^, ycf3^c^, ycf4* |
| **Species** | *Bruguiera sexangula* | |
| **Functions Category** | **Group of Genes** | **Gene Name** |
| **Self-replication** | Small subunit of ribosome | *rps2, rps3, rps4, rps7^a^, rps8, rps11,rps12^acde^, rps14, rps15, rps18, rps19^ad^* |
|  | large subunit of ribosome | *rpl2^ad^, rpl14, rpl16, rpl20,rpl22, rpl23^a^, rpl32,rpl33, rpl36* |
|  | rRNA genes | *rrn4.5^a^, rrn5^a^, rrn16^a^, rrn23^a^* |
|  | DNA-dependent RNA polymerase | *rpoA, rpoB, rpoC1^b^, rpoC2* |
|  | rRNA Genes | *trnY-GUA, trnW-CCA, trnV-UAC^b^, trnV-GAC^a^, trnT-UGU, trnT-GGU, trnS-UGA, trnS-GGA, trnS-GCU, trnR-UCU, trnR-ACG^a^, trnQ-UUG, trnP-UGG, trnN-GUU^a^,trnM-CAU^a^, trnL-UAG, trnL-UAA^b^, trnL-CAA^a^, trnK-UUU^b^, trnI-GAU^ab^, trnI-CAU^a^, trnH-GUG, trnG-GCC^b^, trnG-UCC^a^, trnfM-CAU, trnF-GAA, trnE-UUC, trnD-GUC, trnC-GCA, trnA-UGC^ab^* |
| **Genes for Photosynthesis** | Subunits of ATP synthase | *atpA, atpB, atpE, atpF^b^, atpH, atpI* |
|  | Subunits of NADH-dehydrogenase | *ndhA^b^, ndhB^ab^, ndhC, ndhD, ndhE, ndhF, ndhG, ndhH, ndhI, ndhJ, ndhK* |
|  | Subunits of cytochrome b/f comples | *petA, petB, petD, petG, petL, petN* |
|  | Subunits of photosystem I | *psaA, psaB, psaC, psaI, psaJ* |
|  | Subunits of photosystem II | *psbA, psbB, psbC, psbD, psbE, psbF, psbH, psbI, psbJ, psbK, psbL, psbM, psbN, psbT, psbZ* |
|  | Subunits of rubisco | *rbcL* |
| **Other Genes** | Subunits of Acetyl-CoA-carboxylase | *accD* |
|  | Envelop membrane protein | *cemA* |
|  | c-type cytochrome synthesis gene | *ccsA* |
|  | Protease | *clpP^c^* |
|  | Translational initiation factor |  |
|  | Maturase | *matK* |
|  | Elongation factor |  |
| **Genes of Unknown Function** | Conserved open reading frames | *ycf1^abd^, ycf2^a^, ycf3^c^, ycf4* |
| *Bruguiera × rhynchopetala* | | |
| **Functions Category** | **Group of Genes** | **Gene Name** |
| **Self-replication** | Small subunit of ribosome | *rps2, rps3, rps4, rps7^a^, rps8, rps11,rps12^acde^, rps14, rps15, rps18, rps19^ad^* |
|  | large subunit of ribosome | *rpl2^ad^, rpl14, rpl16^b^, rpl20,rpl22, rpl23^a^, rpl32,rpl33, rpl36* |
|  | rRNA genes | *rrn4.5^a^, rrn5^a^, rrn16^a^, rrn23^a^* |
|  | DNA-dependent RNA polymerase | *rpoA, rpoB, rpoC1^b^, rpoC2* |
|  | rRNA Genes | *trnY-GUA, trnW-CCA, trnV-UAC^b^, trnV-GAC^a^, trnT-UGU, trnT-GGU, trnS-UGA, trnS-GGA, trnS-GCU, trnR-UCU, trnR-ACG^a^, trnQ-UUG, trnP-UGG, trnN-GUU^a^,trnM-CAU, trnL-UAG, trnL-UAA^b^, trnL-CAA^a^, trnK-UUU^b^, trnI-GAU^ab^, trnI-CAU^a^, trnH-GUG, trnG-UCC^a^, trnfM-CAU, trnF-GAA, trnE-UUC, trnD-GUC, trnC-GCA, trnA-UGC^ab^* |
| **Genes for Photosynthesis** | Subunits of ATP synthase | *atpA, atpB, atpE, atpF^b^, atpH, atpI* |
|  | Subunits of NADH-dehydrogenase | *ndhA^b^, ndhB^ab^, ndhC, ndhD, ndhE, ndhF, ndhG, ndhH, ndhI, ndhJ, ndhK* |
|  | Subunits of cytochrome b/f comples | *petA, petB, petD, petG, petL, petN* |
|  | Subunits of photosystem I | *psaA, psaB, psaC, psaI, psaJ* |
|  | Subunits of photosystem II | *psbA, psbB, psbC, psbD, psbE, psbF, psbH, psbI, psbJ, psbK, psbL, psbM, psbN, psbT, psbZ* |
|  | Subunits of rubisco | *rbcL* |
| **Other Genes** | Subunits of Acetyl-CoA-carboxylase | *accD* |
|  | Envelop membrane protein | *cemA* |
|  | c-type cytochrome synthesis gene | *ccsA* |
|  | Protease | *clpP^c^* |
|  | Translational initiation factor |  |
|  | Maturase | *matK* |
|  | Elongation factor |  |
| **Genes of Unknown Function** | Conserved open reading frames | *ycf1^abd^, ycf2^a^, ycf3^c^, ycf4* |
| *Rhizophora apiculata* | | |
| **Functions Category** | **Group of Genes** | **Gene Name** |
| **Self-replication** | Small subunit of ribosome | *rps2, rps3, rps4, rps7^a^, rps8, rps11,rps12^acde^, rps14, rps15, rps18, rps19* |
|  | large subunit of ribosome | *rpl2^ad^, rpl14, rpl16^b^, rpl20,rpl22, rpl23^a^, rpl33, rpl36* |
|  | rRNA genes | *rrn4.5^a^, rrn5^a^, rrn16^a^, rrn23^a^* |
|  | DNA-dependent RNA polymerase | *rpoA, rpoB, rpoC1^b^, rpoC2* |
|  | rRNA Genes | *trnY-GUA, trnW-CCA, trnV-UAC^b^, trnV-GAC^a^, trnT-UGU, trnT-GGU, trnS-UGA, trnS-GGA, trnS-GCU, trnR-UCU, trnR-ACG^a^, trnQ-UUG, trnP-UGG, trnN-GUU^a^,trnM-CAU, trnL-UAG, trnL-UAA^b^, trnL-CAA, trnK-UUU^b^, trnI-GAU^ab^, trnI-CAU^a^, trnH-GUG, trnG-GCC^b^, trnG-UCC, trnfM-CAU, trnF-GAA, trnE-UUC, trnD-GUC, trnC-GCA, trnA-UGC^ab^* |
| **Genes for Photosynthesis** | Subunits of ATP synthase | *atpA, atpB, atpE, atpF^b^, atpH, atpI* |
|  | Subunits of NADH-dehydrogenase | *ndhA^b^, ndhB^ab^, ndhC, ndhD, ndhE, ndhF, ndhG, ndhH, ndhI, ndhJ, ndhK* |
|  | Subunits of cytochrome b/f comples | *petA, petB^b^, petD^b^, petG, petL, petN* |
|  | Subunits of photosystem I | *psaA, psaB, psaC, psaI, psaJ* |
|  | Subunits of photosystem II | *psbA, psbB, psbC, psbD, psbE, psbF, psbH, psbI, psbJ, psbK, psbL, psbM, psbN, psbT, psbZ* |
|  | Subunits of rubisco | *rbcL* |
| **Other Genes** | Subunits of Acetyl-CoA-carboxylase | *accD* |
|  | Envelop membrane protein | *cemA* |
|  | c-type cytochrome synthesis gene | *ccsA* |
|  | Protease | *clpP^c^* |
|  | Translational initiation factor |  |
|  | Maturase | *matK* |
|  | Elongation factor |  |
| **Genes of Unknown Function** | Conserved open reading frames | *ycf1^ab^, ycf2^a^, ycf3^c^, ycf4, ycf15* |
| *Rhizophora stylosa* | | |
| **Functions Category** | **Group of Genes** | **Gene Name** |
| **Self-replication** | Small subunit of ribosome | *rps2, rps3, rps4, rps7^a^, rps8, rps11,rps12^acde^, rps14, rps15, rps18, rps19* |
|  | large subunit of ribosome | *rpl2^ad^, rpl14, rpl16^b^, rpl20,rpl22, rpl23^a^, rpl33, rpl36* |
|  | rRNA genes | *rrn4.5^a^, rrn5^a^, rrn16^a^, rrn23^a^* |
|  | DNA-dependent RNA polymerase | *rpoA, rpoB, rpoC1^b^, rpoC2* |
|  | rRNA Genes | *trnY-GUA, trnW-CCA, trnV-UAC^b^, trnV-GAC^a^, trnT-UGU, trnT-GGU, trnS-UGA, trnS-GGA, trnS-GCU, trnR-UCU, trnR-ACG^a^, trnQ-UUG, trnP-UGG, trnN-GUU^a^,trnM-CAU, trnL-UAG, trnL-UAA^b^, trnL-CAA^a^, trnK-UUU^b^, trnI-GAU^ab^, trnI-CAU^a^, trnH-GUG, trnG-GCC, trnG-UCC, trnfM-CAU, trnF-GAA, trnE-UUC, trnD-GUC, trnC-GCA, trnA-UGC^ab^* |
| **Genes for Photosynthesis** | Subunits of ATP synthase | *atpA, atpB, atpE, atpF^b^, atpH, atpI* |
|  | Subunits of NADH-dehydrogenase | *ndhA^b^, ndhB^ab^, ndhC, ndhD, ndhE, ndhF, ndhG, ndhH, ndhI, ndhJ, ndhK* |
|  | Subunits of cytochrome b/f comples | *petA, petB^b^, petD^b^, petG, petL, petN* |
|  | Subunits of photosystem I | *psaA, psaB, psaC, psaI, psaJ* |
|  | Subunits of photosystem II | *psbA, psbB, psbC, psbD, psbE, psbF, psbH, psbI, psbJ, psbK, psbL, psbM, psbN, psbT, psbZ* |
|  | Subunits of rubisco | *rbcL* |
| **Other Genes** | Subunits of Acetyl-CoA-carboxylase | *accD* |
|  | Envelop membrane protein | *cemA* |
|  | c-type cytochrome synthesis gene | *ccsA* |
|  | Protease | *clpP^c^* |
|  | Translational initiation factor |  |
|  | Maturase | *matK* |
|  | Elongation factor |  |
| **Genes of Unknown Function** | Conserved open reading frames | *ycf1^ab^, ycf2^a^, ycf3^c^, ycf4* |
| *Rhizophora× lamarkii* | | |
| **Functions Category** | **Group of Genes** | **Gene Name** |
| **Self-replication** | Small subunit of ribosome | *rps2, rps3, rps4, rps7^a^, rps8, rps11,rps12^acde^, rps14, rps15, rps18, rps19^ad^* |
|  | large subunit of ribosome | *rpl2^ad^, rpl14, rpl16^b^, rpl20,rpl22, rpl23^a^, rpl32,rpl33, rpl36* |
|  | rRNA genes | *rrn4.5^a^, rrn5^a^, rrn16^a^, rrn23^a^* |
|  | DNA-dependent RNA polymerase | *rpoA, rpoB, rpoC1^b^, rpoC2* |
|  | rRNA Genes | *trnY-GUA, trnW-CCA, trnV-UAC^b^, trnV-GAC^a^, trnT-UGU, trnT-GGU, trnS-UGA, trnS-GGA, trnS-GCU, trnR-UCU, trnR-ACG^a^, trnQ-UUG, trnP-UGG, trnN-GUU^a^,trnM-CAU, trnL-UAG, trnL-UAA^b^, trnL-CAA^a^, trnK-UUU^b^, trnI-GAU^ab^, trnI-CAU^a^, trnH-GUG, trnG-UCC^a^, trnfM-CAU, trnF-GAA, trnE-UUC, trnD-GUC, trnC-GCA, trnA-UGC^ab^* |
| **Genes for Photosynthesis** | Subunits of ATP synthase | *atpA, atpB, atpE, atpF^b^, atpH, atpI* |
|  | Subunits of NADH-dehydrogenase | *ndhA^b^, ndhB^ab^, ndhC, ndhD, ndhE, ndhF, ndhG, ndhH, ndhI, ndhJ, ndhK* |
|  | Subunits of cytochrome b/f comples | *petA, petB^b^, petD^b^, petG, petL, petN* |
|  | Subunits of photosystem I | *psaA, psaB, psaC, psaI, psaJ* |
|  | Subunits of photosystem II | *psbA, psbB, psbC, psbD, psbE, psbF, psbH, psbI, psbJ, psbK, psbL, psbM, psbN, psbT, psbZ* |
|  | Subunits of rubisco | *rbcL* |
| **Other Genes** | Subunits of Acetyl-CoA-carboxylase | *accD* |
|  | Envelop membrane protein | *cemA* |
|  | c-type cytochrome synthesis gene | *ccsA* |
|  | Protease | *clpP^c^* |
|  | Translational initiation factor |  |
|  | Maturase | *matK* |
|  | Elongation factor |  |
| **Genes of Unknown Function** | Conserved open reading frames | *ycf1^ad^, ycf2^a^, ycf3^c^, ycf4* |
| *Ceriops tagal* | | |
| **Functions Category** | **Group of Genes** | **Gene Name** |
| **Self-replication** | Small subunit of ribosome | *rps2, rps3, rps4, rps7^a^, rps8, rps11,rps12^acde^, rps14, rps15, rps18, rps19^ac^* |
|  | large subunit of ribosome | *rpl2^ad^, rpl14, rpl16, rpl20,rpl22^d^, rpl23^a^, rpl32,rpl33, rpl36* |
|  | rRNA genes | *rrn4.5^a^, rrn5^a^, rrn16^a^, rrn23^a^* |
|  | DNA-dependent RNA polymerase | *rpoA, rpoB, rpoC1^b^, rpoC2* |
|  | rRNA Genes | *trnY-GUA, trnW-CCA, trnV-UAC^b^, trnV-GAC^a^, trnT-UGU, trnT-GGU, trnS-UGA, trnS-GGA, trnS-GCU, trnR-UCU, trnR-ACG^a^, trnQ-UUG, trnP-GGG, trnP-UGG, trnN-GUU^a^,trnM-CAU^a^, trnL-UAG, trnL-UAA^b^, trnL-CAA^a^, trnK-UUU, trnI-GAU^ab^, trnI-CAU^a^, trnH-GUG, trnG-UCC, trnfM-CAU, trnF-GAA, trnE-UUC, trnD-GUC, trnC-GCA, trnA-UGC^ab^* |
| **Genes for Photosynthesis** | Subunits of ATP synthase | *atpA, atpB, atpE, atpF^b^, atpH, atpI* |
|  | Subunits of NADH-dehydrogenase | *ndhA^b^, ndhB^ab^, ndhC, ndhD, ndhE, ndhF, ndhG, ndhH, ndhI, ndhJ, ndhK* |
|  | Subunits of cytochrome b/f comples | *petA, petB^b^, petD^b^, petG, petL, petN* |
|  | Subunits of photosystem I | *psaA, psaB, psaC, psaI, psaJ* |
|  | Subunits of photosystem II | *psbA, psbB, psbC, psbD, psbE, psbF, psbH, psbI, psbJ, psbK, psbL, psbM, psbN, psbT, psbZ* |
|  | Subunits of rubisco | *rbcL* |
| **Other Genes** | Subunits of Acetyl-CoA-carboxylase | *accD* |
|  | Envelop membrane protein | *cemA* |
|  | c-type cytochrome synthesis gene | *ccsA* |
|  | Protease | *clpP^c^* |
|  | Translational initiation factor |  |
|  | Maturase | *matK* |
|  | Elongation factor |  |
| **Genes of Unknown Function** | Conserved open reading frames | *ycf1^ac^, ycf2^a^, ycf3^c^, ycf4* |
|  |  | *Kandelia obvata* ([Yang et al., 2019](#_ENREF_1)) |
| **Functions Category** | **Group of Genes** | **Gene Name** |
| **Self-replication** | Small subunit of ribosome | *rps2, rps3, rps4, rps7^a^, rps8, rps11,rps12^acde^, rps14, rps15, rps18, rps19^a^* |
|  | large subunit of ribosome | *rpl2^ad^, rpl14, rpl16^b^, rpl20,rpl22, rpl23^a^, rpl32,rpl33, rpl36* |
|  | rRNA genes | *rrn4.5^a^, rrn5^a^, rrn16^a^, rrn23^a^* |
|  | DNA-dependent RNA polymerase | *rpoA, rpoB, rpoC1^b^, rpoC2* |
|  | rRNA Genes | *trnY-GUA, trnW-CCA, trnV-UAC^b^, trnV-GAC^a^, trnT-UGU, trnT-GGU, trnS-UGA, trnS-GGA, trnS-GCU, trnR-UCU, trnR-ACG^a^, trnQ-UUG, trnP-UGG, trnN-GUU^a^, trnM-CAU^a^, trnL-UAG, trnL-UAA^b^ trnL-CAA^a^, trnK-UUU^b^, trnI-GAU^ab^, trnI-CAU^a^, trnH-GUG, trnG-UCC, trnfM-CAU, trnF-GAA, trnE-UUC, trnD-GUC, trnC-GCA, trnA-UGC^ab^* |
| **Genes for Photosynthesis** | Subunits of ATP synthase | *atpA, atpB, atpE, atpF^b^, atpH, atpI* |
|  | Subunits of NADH-dehydrogenase | *ndhA^b^, ndhB^ab^, ndhC, ndhD, ndhE, ndhG, ndhH, ndhI, ndhJ, ndhK* |
|  | Subunits of cytochrome b/f comples | *petA, petB^b^, petD^b^, petG, petL, petN* |
|  | Subunits of photosystem I | *psaA, psaB, psaC, psaI, psaJ* |
|  | Subunits of photosystem II | *psbA, psbB, psbC, psbD, psbE, psbF, psbH, psbJ, psbK, psbL, psbM, psbN, psbT, psbZ* |
|  | Subunits of rubisco | *rbcL* |
| **Other Genes** | Subunits of Acetyl-CoA-carboxylase | *accD* |
|  | Envelop membrane protein | *cemA* |
|  | c-type cytochrome synthesis gene | *ccsA* |
|  | Protease | *clpP^c^* |
|  | Translational initiation factor |  |
|  | Maturase | *matK* |
|  | Elongation factor |  |
| **Genes of Unknown Function** | Conserved open reading frames | *ycf1^abd^, ycf2^a^, ycf3^c^, ycf4* |

a—Two gene copies in inverted repeat (IRs); b—Gene containing a single intron; c—Gene containing two introns; d—Pseudogene; e—Gene divided into two independent transcription units.

**Table S3 SSR numbers in eight Rhizophoracea mangrove species.**

|  | *B. gymnorrhiza* | *B. sexangula* | *B. × rhynchopetala* | *R. apiculata* | *R. stylosa* | *R. × lamarkii* | *C. tagal* | *K. obvata* |
| --- | --- | --- | --- | --- | --- | --- | --- | --- |
| Mono | 114 | 112 | 114 | 112 | 120 | 117 | 79 | 92 |
| Di | 34 | 38 | 36 | 20 | 18 | 22 | 16 | 29 |
| Tri | 13 | 14 | 13 | 8 | 9 | 13 | 20 | 17 |
| Tetra | 20 | 19 | 20 | 8 | 19 | 10 | 22 | 18 |
| Penta | 6 | 7 | 6 | 7 | 9 | 9 | 4 | 8 |
| Hexa | 1 | 1 | 1 | 1 | 3 | 2 | 1 | 0 |
| Sum | 188 | 191 | 190 | 156 | 178 | 173 | 142 | 164 |

**Table S4 SSR types in eight Rhizophoraceae mangrove species.**

|  | *B. gymnorrhiza* | *B. sexangula* | *B. × rhynchopetala* | *R. apiculata* | *R. stylosa* | *R. × lamarkii* | *C. tagal* | *K. obvata* |
| --- | --- | --- | --- | --- | --- | --- | --- | --- |
| A/T | 107 | 109 | 107 | 106 | 118 | 113 | 76 | 92 |
| C/G | 7 | 3 | 7 | 6 | 2 | 4 | 3 | 3 |
| AG/CT | 4 | 4 | 4 | 2 | 2 | 2 | 2 | 26 |
| AT/AT | 30 | 34 | 32 | 18 | 16 | 20 | 14 | 1 |
| AAG/CTT | 0 | 0 | 0 | 0 | 0 | 0 | 1 | 0 |
| AGG/CTT | 0 | 0 | 0 | 0 | 0 | 0 | 0 | 1 |
| AAT/AAT | 13 | 14 | 13 | 8 | 9 | 13 | 19 | 15 |
| AAAG/CTTT | 5 | 5 | 5 | 1 | 3 | 2 | 7 | 3 |
| AAAT/ATTT | 10 | 9 | 10 | 5 | 14 | 7 | 10 | 12 |
| AATC/ATTG | 1 | 1 | 1 | 1 | 1 | 1 | 1 | 1 |
| AATT/AATT | 2 | 2 | 2 | 1 | 1 | 0 | 0 | 1 |
| AATG/ATTC | 0 | 0 | 0 | 1 | 0 | 0 | 0 | 0 |
| AGAT/ATCT | 1 | 1 | 1 | 0 | 0 | 0 | 4 | 0 |
| ATCC/ATGG | 1 | 1 | 1 | 0 | 0 | 0 | 0 | 0 |
| AAAAG/CTTTT | 0 | 0 | 0 | 1 | 1 | 1 | 1 | 0 |
| AAAAT/ATTTT | 2 | 3 | 2 | 1 | 1 | 1 | 1 | 3 |
| AAACT/AGTTT | 1 | 1 | 1 | 0 | 0 | 0 | 0 | 0 |
| AAATC/ATTTG | 1 | 1 | 1 | 1 | 1 | 1 | 0 | 1 |
| AATAT/ATATT | 1 | 2 | 1 | 1 | 1 | 3 | 0 | 0 |
| AATGA/ATTGC | 0 | 0 | 0 | 2 | 0 | 0 | 0 | 0 |
| AATGC/ATTGC | 0 | 0 | 0 | 2 | 2 | 1 | 1 | 2 |
| AATTC/AATTG | 1 | 0 | 1 | 0 | 0 | 0 | 0 | 0 |
| ACTAT/AGTAT | 0 | 0 | 0 | 1 | 0 | 0 | 0 | 0 |
| AATAC/ATTGT | 0 | 0 | 0 | 0 | 0 | 0 | 0 | 1 |
| AAAATT/AATTTT | 0 | 0 | 0 | 0 | 0 | 1 | 0 | 0 |
| ACCTAT/AGGTAT | 1 | 1 | 1 | 0 | 0 | 0 | 0 | 0 |
| ATATCC/ATATGG | 0 | 0 | 0 | 1 | 0 | 1 | 1 | 0 |
| AATATT/AATATT | 0 | 0 | 0 | 0 | 1 | 0 | 0 | 0 |
| AATTAT/AATTAT | 0 | 0 | 0 | 0 | 1 | 0 | 0 | 0 |

**Table S5 RNA editing sites predicted by the PREP program in eight Rhizophoraceae mangroves.**

|  |  | *B. gymnorrhiza* | *B. sexangula* | *B. × rhynchopetala* | *R. apiculata* | *R. stylosa* | *R. × lamarkii* | *C. tagal* | *K. obvata* |
| --- | --- | --- | --- | --- | --- | --- | --- | --- | --- |
| Gene | A.A position | Codon(A.A) conversion |  |  |  |  |  |  |  |
| accD | 269 | TCG (S) => TTG (L) | TCG (S) => TTG (L) | TCG (S) => TTG (L) | TCG (S) => TTG (L) | TCG (S) => TTG (L) | TCG (S) => TTG (L) | TCG (S) => TTG (L) | TCG (S) => TTG (L) |
|  | 472 | CCT (P) => CTT (L) | CCT (P) => CTT (L) | CCT (P) => CTT (L) | CCT (P) => CTT (L) | CCT (P) => CTT (L) | CCT (P) => CTT (L) | CCT (P) => CTT (L) | CCT (P) => CTT (L) |
|  | 496 | ACT (T) => ATT (I) | ACT (T) => ATT (I) | ACT (T) => ATT (I) |  |  |  |  |  |
| atpB |  |  |  |  | GCA (A) => GTA (V) | GCA (A) => GTA (V) | GCA (A) => GTA (V) |  |  |
| atpF | 31 | CCA (P) => CTA (L) | CCA (P) => CTA (L) | CCA (P) => CTA (L) | CCA (P) => CTA (L) | CCA (P) => CTA (L) | CCA (P) => CTA (L) | CCA (P) => CTA (L) | CCA (P) => CTA (L) |
| atpI | 210 | TCA (S) => TTA (L) | TCA (S) => TTA (L) | TCA (S) => TTA (L) | TCA (S) => TTA (L) | TCA (S) => TTA (L) | TCA (S) => TTA (L) | TCA (S) => TTA (L) | TCA (S) => TTA (L) |
| clpP | 187 | CAT (H) => TAT (Y) | CAT (H) => TAT (Y) | CAT (H) => TAT (Y) | CAT (H) => TAT (Y) | CAT (H) => TAT (Y) | CAT (H) => TAT (Y) | CAT (H) => TAT (Y) | CAT (H) => TAT (Y) |
| matK | 178 | CTC (L) => TTC (F) | CTC (L) => TTC (F) | CTC (L) => TTC (F) | CTC (L) => TTC (F) | CTC (L) => TTC (F) | CTC (L) => TTC (F) | CTC (L) => TTC (F) | CTC (L) => TTC (F) |
|  | 214 | CAT (H) => TAT (Y) | CAT (H) => TAT (Y) | CAT (H) => TAT (Y) |  |  |  | CAT (H) => TAT (Y) |  |
|  | 392 | CGG (R) => TGG (W) | CGG (R) => TGG (W) | CGG (R) => TGG (W) | CGG (R) => TGG (W) | CGG (R) => TGG (W) | CGG (R) => TGG (W) | CGG (R) => TGG (W) | CGG (R) => TGG (W) |
|  | 396 | TCA (S) => TTA (L) | TCA (S) => TTA (L) | TCA (S) => TTA (L) | TCA (S) => TTA (L) | TCA (S) => TTA (L) | TCA (S) => TTA (L) | TCA (S) => TTA (L) | TCA (S) => TTA (L) |
|  | 501 | CCG (P) => CTG (L) | CCG (P) => CTG (L) | CCG (P) => CTG (L) | CCG (P) => CTG (L) | CCG (P) => CTG (L) | CCG (P) => CTG (L) | CCG (P) => CTG (L) |  |
| ndhA |  |  |  |  |  | ACA (T) => ATA (I) | ACA (T) => ATA (I) |  |  |
|  | 81 | ACT (T) => ATT (I) | ACT (T) => ATT (I) | ACT (T) => ATT (I) | ACT (T) => ATT (I) | ACT (T) => ATT (I) | ACT (T) => ATT (I) |  | ACT (T) => ATT (I) |
|  | 86 | TCG (S) => TTG (L) | TCG (S) => TTG (L) | TCG (S) => TTG (L) |  |  |  |  | TCG (S) => TTG (L) |
|  | 161 | TCA (S) => TTA (L) | TCA (S) => TTA (L) | TCA (S) => TTA (L) |  |  |  |  | TCA (S) => TTA (L) |
|  | 330 | TCC (S) => TTC (F) | TCC (S) => TTC (F) | TCC (S) => TTC (F) | TCC (S) => TTC (F) | TCC (S) => TTC (F) | TCC (S) => TTC (F) |  | TCC (S) => TTC (F) |
| ndhB | 50 | TCA (S) => TTA (L) | TCA (S) => TTA (L) | TCA (S) => TTA (L) | TCA (S) => TTA (L) | TCA (S) => TTA (L) | TCA (S) => TTA (L) | TCA (S) => TTA (L) | TCA (S) => TTA (L) |
|  | 156 | CCA (P) => CTA (L) | CCA (P) => CTA (L) | CCA (P) => CTA (L) | CCA (P) => CTA (L) | CCA (P) => CTA (L) | CCA (P) => CTA (L) | CCA (P) => CTA (L) | CCA (P) => CTA (L) |
|  | 196 | CAT (H) => TAT (Y) | CAT (H) => TAT (Y) | CAT (H) => TAT (Y) | CAT (H) => TAT (Y) | CAT (H) => TAT (Y) | CAT (H) => TAT (Y) | CAT (H) => TAT (Y) | CAT (H) => TAT (Y) |
|  | 204 | TCG (S) => TTG (L) | TCG (S) => TTG (L) | TCG (S) => TTG (L) | TCG (S) => TTG (L) | TCG (S) => TTG (L) | TCG (S) => TTG (L) | TCG (S) => TTG (L) | TCG (S) => TTG (L) |
|  | 246 | CCA (P) => CTA (L) | CCA (P) => CTA (L) | CCA (P) => CTA (L) | CCA (P) => CTA (L) | CCA (P) => CTA (L) | CCA (P) => CTA (L) | CCA (P) => CTA (L) | CCA (P) => CTA (L) |
|  | 249 | TCT (S) => TTT (F) | TCT (S) => TTT (F) | TCT (S) => TTT (F) | TCT (S) => TTT (F) | TCT (S) => TTT (F) | TCT (S) => TTT (F) | TCT (S) => TTT (F) | TCT (S) => TTT (F) |
|  | 277 | TCA (S) => TTA (L) | TCA (S) => TTA (L) | TCA (S) => TTA (L) | TCA (S) => TTA (L) | TCA (S) => TTA (L) | TCA (S) => TTA (L) | TCA (S) => TTA (L) | TCA (S) => TTA (L) |
|  | 279 | TCA (S) => TTA (L) | TCA (S) => TTA (L) | TCA (S) => TTA (L) | TCA (S) => TTA (L) | TCA (S) => TTA (L) | TCA (S) => TTA (L) | TCA (S) => TTA (L) | TCA (S) => TTA (L) |
|  | 419 | CAT (H) => TAT (Y) | CAT (H) => TAT (Y) | CAT (H) => TAT (Y) | CAT (H) => TAT (Y) | CAT (H) => TAT (Y) | CAT (H) => TAT (Y) | CAT (H) => TAT (Y) | CAT (H) => TAT (Y) |
|  | 494 | CCA (P) => CTA (L) | CCA (P) => CTA (L) | CCA (P) => CTA (L) | CCA (P) => CTA (L) | CCA (P) => CTA (L) | CCA (P) => CTA (L) | CCA (P) => CTA (L) | CCA (P) => CTA (L) |
| ndhD | 1 | ACG (T) => ATG (M) |  |  | ACG (T) => ATG (M) | ACG (T) => ATG (M) |  |  | ACG (T) => ATG (M) |
|  | 16 | TCC (S) => TTC (F) |  |  | TCC (S) => TTC (F) | TCC (S) => TTC (F) | TCC (S) => TTC (F) |  | TCC (S) => TTC (F) |
|  | 182 | GCT (A) => GTT (V) | GCT (A) => GTT (V) | GCT (A) => GTT (V) | GCT (A) => GTT (V) | GCT (A) => GTT (V) | GCT (A) => GTT (V) | GCT (A) => GTT (V) | GCT (A) => GTT (V) |
|  | 293 | TCA (S) => TTA (L) | TCA (S) => TTA (L) | TCA (S) => TTA (L) | TCA (S) => TTA (L) | TCA (S) => TTA (L) | TCA (S) => TTA (L) | TCA (S) => TTA (L) | TCA (S) => TTA (L) |
|  | 359 | GCT (A) => GTT (V) | GCT (A) => GTT (V) | GCT (A) => GTT (V) | GCT (A) => GTT (V) | GCT (A) => GTT (V) | GCT (A) => GTT (V) | GCT (A) => GTT (V) | GCT (A) => GTT (V) |
|  | 369 | ACC (T) => ATC (I) | ACC (T) => ATC (I) | ACC (T) => ATC (I) | ACC (T) => ATC (I) | ACC (T) => ATC (I) | ACC (T) => ATC (I) | ACC (T) => ATC (I) | ACC (T) => ATC (I) |
|  | 469 | CTT (L) => TTT (F) | CTT (L) => TTT (F) | CTT (L) => TTT (F) | CTT (L) => TTT (F) | CTT (L) => TTT (F) | CTT (L) => TTT (F) | CTT (L) => TTT (F) | CTT (L) => TTT (F) |
| ndhF | 87 | CAC (H) => TAC (Y) | CAC (H) => TAC (Y) | CAC (H) => TAC (Y) | CAC (H) => TAC (Y) | CAC (H) => TAC (Y) | CAC (H) => TAC (Y) | CAC (H) => TAC (Y) | CAC (H) => TAC (Y) |
|  | 97 | TCA (S) => TTA (L) | TCA (S) => TTA (L) | TCA (S) => TTA (L) | TCA (S) => TTA (L) | TCA (S) => TTA (L) | TCA (S) => TTA (L) | TCA (S) => TTA (L) | TCA (S) => TTA (L) |
|  | 105 | ACT (T) => ATT (I) | ACT (T) => ATT (I) | ACT (T) => ATT (I) | ACT (T) => ATT (I) | ACT (T) => ATT (I) | ACT (T) => ATT (I) | ACT (T) => ATT (I) | ACT (T) => ATT (I) |
|  | 196 | CTT (L) => TTT (F) | CTT (L) => TTT (F) | CTT (L) => TTT (F) | CTT (L) => TTT (F) | CTT (L) => TTT (F) | CTT (L) => TTT (F) | CTT (L) => TTT (F) | CTT (L) => TTT (F) |
|  | 478 | CCA (P) => CTA (L) | CCA (P) => CTA (L) | CCA (P) => CTA (L) | CCA (P) => CTA (L) | CCA (P) => CTA (L) | CCA (P) => CTA (L) | CCA (P) => CTA (L) | CCA (P) => CTA (L) |
|  | 643 | CTT (L) => TTT (F) | CTT (L) => TTT (F) | CTT (L) => TTT (F) | CTT (L) => TTT (F) | CTT (L) => TTT (F) | CTT (L) => TTT (F) | CTT (L) => TTT (F) | CTT (L) => TTT (F) |
|  | 709 | CTT (L) => TTT (F) | CTT (L) => TTT (F) | CTT (L) => TTT (F) | CTT (L) => TTT (F) | CTT (L) => TTT (F) | CTT (L) => TTT (F) | CTT (L) => TTT (F) | CTT (L) => TTT (F) |
| ndhG | 56 | CAT (H) => TAT (Y) | CAT (H) => TAT (Y) | CAT (H) => TAT (Y) | CAT (H) => TAT (Y) | CAT (H) => TAT (Y) | CAT (H) => TAT (Y) | CAT (H) => TAT (Y) |  |
|  | 105 | ACA (T) => ATA (I) | ACA (T) => ATA (I) | ACA (T) => ATA (I) | ACA (T) => ATA (I) | ACA (T) => ATA (I) | ACA (T) => ATA (I) | ACA (T) => ATA (I) | ACA (T) => ATA (I) |
| petB | 204 | CCA (P) => CTA (L) | CCA (P) => CTA (L) | CCA (P) => CTA (L) | CCA (P) => CTA (L) | CCA (P) => CTA (L) | CCA (P) => CTA (L) | CCA (P) => CTA (L) | CCA (P) => CTA (L) |
| psaI | 28 | TCT (S) => TTT (F) | TCT (S) => TTT (F) | TCT (S) => TTT (F) | TCT (S) => TTT (F) | TCT (S) => TTT (F) | TCT (S) => TTT (F) | TCT (S) => TTT (F) | TCT (S) => TTT (F) |
| psbF | 26 | TCT (S) => TTT (F) | TCT (S) => TTT (F) | TCT (S) => TTT (F) |  |  |  |  | TCT (S) => TTT (F) |
| rpl2 | 199 | GCT (A) => GTT (V) | GCT (A) => GTT (V) | GCT (A) => GTT (V) | GCT (A) => GTT (V) | GCT (A) => GTT (V) | GCT (A) => GTT (V) | GCT (A) => GTT (V) | GCT (A) => GTT (V) |
| rpoA | 296 | TCA (S) => TTA (L) | TCT (S) => TTT (F) | TCT (S) => TTT (F) | TCA (S) => TTA (L) | TCA (S) => TTA (L) | TCA (S) => TTA (L) | TCA (S) => TTA (L) | TCA (S) => TTA (L) |
|  | 332 | TCG (S) => TTG (L) | TCA (S) => TTA (L) | TCA (S) => TTA (L) | TCA (S) => TTA (L) | TCA (S) => TTA (L) | TCA (S) => TTA (L) | TCG (S) => TTG (L) |  |
|  |  |  |  |  | TCA (S) => TTA (L) | TCA (S) => TTA (L) | TCA (S) => TTA (L) |  |  |
| rpoB | 113 | TCT (S) => TTT (F) | TCT (S) => TTT (F) | TCT (S) => TTT (F) | TCT (S) => TTT (F) | TCT (S) => TTT (F) | TCT (S) => TTT (F) |  | TCT (S) => TTT (F) |
|  | 184 | TCA (S) => TTA (L) | TCA (S) => TTA (L) | TCA (S) => TTA (L) | TCA (S) => TTA (L) | TCA (S) => TTA (L) | TCA (S) => TTA (L) | TCA (S) => TTA (L) | TCA (S) => TTA (L) |
|  | 189 | TCA (S) => TTA (L) | TCA (S) => TTA (L) | TCA (S) => TTA (L) | TCA (S) => TTA (L) | TCA (S) => TTA (L) | TCA (S) => TTA (L) | TCA (S) => TTA (L) | TCA (S) => TTA (L) |
|  |  |  |  |  |  |  |  |  | CAT (H) => TAT (Y) |
| rpoC1 | 14 | TCA (S) => TTA (L) | TCA (S) => TTA (L) | TCA (S) => TTA (L) | TCA (S) => TTA (L) | TCA (S) => TTA (L) | TCA (S) => TTA (L) | TCA (S) => TTA (L) | TCA (S) => TTA (L) |
|  |  |  |  |  | ACC (T) => ATC (I) | ACC (T) => ATC (I) | ACC (T) => ATC (I) |  |  |
|  | 483 | CGT (R) => TGT (C) | CGT (R) => TGT (C) | CGT (R) => TGT (C) | CGT (R) => TGT (C) | CGT (R) => TGT (C) | CGT (R) => TGT (C) | CGT (R) => TGT (C) | CGT (R) => TGT (C) |
|  |  |  |  |  |  |  |  | CCT (P) => CTT (L) |  |
| rpoC2 | 484 | CGC (R) => TGC (C) | CGC (R) => TGC (C) | CGC (R) => TGC (C) | CGC (R) => TGC (C) | CGC (R) => TGC (C) | CGC (R) => TGC (C) | CGC (R) => TGC (C) | CGC (R) => TGC (C) |
|  | 535 | CCT (P) => TTT (F) | CCT (P) => TTT (F) | CCT (P) => TTT (F) | CCT (P) => TTT (F) | CCT (P) => TTT (F) | CCT (P) => TTT (F) | CCT (P) => TTT (F) | CCT (P) => TTT (F) |
|  | 535 | CCT (P) => TTT (F) | CCT (P) => TTT (F) | CCT (P) => TTT (F) | CCT (P) => TTT (F) | CCT (P) => TTT (F) | CCT (P) => TTT (F) | CCT (P) => TTT (F) | CCT (P) => TTT (F) |
|  | 768 | GCC (A) => GTC (V) | GCC (A) => GTC (V) | GCC (A) => GTC (V) | GCC (A) => GTC (V) | GCC (A) => GTC (V) | GCC (A) => GTC (V) | GCC (A) => GTC (V) | GCC (A) => GTC (V) |
|  | 770 | CGG (R) => TGG (W) | CGG (R) => TGG (W) | CGG (R) => TGG (W) | CGG (R) => TGG (W) | CGG (R) => TGG (W) | CGG (R) => TGG (W) | CGG (R) => TGG (W) |  |
|  | 1250 | TCG (S) => TTG (L) | TCG (S) => TTG (L) | TCG (S) => TTG (L) | TCG (S) => TTG (L) | TCG (S) => TTG (L) | TCG (S) => TTG (L) |  | TCG (S) => TTG (L) |
| rps14 | 27 | TCA (S) => TTA (L) | TCA (S) => TTA (L) | TCA (S) => TTA (L) | TCA (S) => TTA (L) | TCA (S) => TTA (L) | TCA (S) => TTA (L) | TCA (S) => TTA (L) | TCA (S) => TTA (L) |
|  | 50 | CCA (P) => CTA (L) | CCA (P) => CTA (L) | CCA (P) => CTA (L) | CCA (P) => CTA (L) | CCA (P) => CTA (L) | CCA (P) => CTA (L) | CCA (P) => CTA (L) | CCA (P) => CTA (L) |
| Total | Sites | 60 | 60 | 60 | 60 | 61 | 60 | 57 | 58 |
|  | First codon | 17 | 16 | 17 | 18 | 17 | 17 | 16 | 15 |
|  | Second codon | 43 | 44 | 43 | 42 | 44 | 43 | 41 | 43 |

**Fig. S1.** Phylogenetic tree of eight Rhizophoraceae mangrove species.
